# The dynamin-like protein Fzl promotes thylakoid fusion and resistance to light stress in *Chlamydomonas reinhardtii*

**DOI:** 10.1371/journal.pgen.1008047

**Published:** 2019-03-15

**Authors:** Justin Findinier, Cédric Delevoye, Mickael M. Cohen

**Affiliations:** 1 Sorbonne Université, CNRS, UMR8226, Institut de Biologie Physico-Chimique, Laboratoire de Biologie Moléculaire et Cellulaire des Eucaryotes, Paris, France; 2 Structure and Membrane Compartments, Institut Curie, Paris Sciences & Lettres Research University, Centre National de la Recherche Scientifique, UMR144, Paris, France; 3 Cell and Tissue Imaging Facility, Institut Curie, Paris Sciences & Lettres Research University, Centre National de la Recherche Scientifique, UMR144, Paris, France; Michigan State University, UNITED STATES

## Abstract

Large GTPases of the Dynamin Related Proteins (DRP) family shape lipid bilayers through membrane fission or fusion processes. Despite the highly organized photosynthetic membranes of thylakoids, a single DRP is known to be targeted inside the chloroplast. Fzl from the land plant *Arabidopsis thaliana* is inserted in the inner envelope and thylakoid membranes to regulate their morphology. Fzl may promote the fusion of thylakoids but this remains to be proven. Moreover, the physiological requirement for fusing thylakoids is currently unknown. Here, we find that the unicellular microalga *Chlamydomonas reinhardtii* encodes an Fzl ortholog (CrFzl) that is localized in the chloroplast where it is soluble. To explore its function, the CRISPR/Cas9 technology was employed to generate multiple CrFzl knock out strains. Phenotypic analyzes revealed a specific requirement of CrFzl for survival upon light stress. Consistent with this, strong irradiance lead to increased photoinhibition of photosynthesis in mutant cells. Fluorescence and electron microscopy analysis demonstrated that upon exposure to high light, CrFzl mutants show defects in chloroplast morphology but also large cytosolic vacuoles in close contact with the plastid. We further observe that strong irradiance induces an increased recruitment of the DRP to thylakoid membranes. Most importantly, we show that CrFzl is required for the fusion of thylakoids during mating. Together, our results suggest that thylakoids fusion may be necessary for resistance to light stress.

## Author summary

All eukaryotic cells are composed of compartments with defined functions. Among those, mitochondria generate the main source of energy in human and animal cells. Their capacity to generate and diffuse energy in the cell is regulated by fusion and fragmentation processes.

Together with mitochondria that produce energy from oxygen, plant cells include an additional compartment called the chloroplast that produces energy from light. The machinery that converts light into energy is more precisely located inside the chloroplast within stacks of membranes called the thylakoids. Here, we elucidate the function of CrFzl, a previously uncharacterized protein encoded by the genome of the unicellular alga *Chlamydomonas reinhardtii*. Algal cells that do not contain CrFzl are impaired in their capacity to grow when they receive too much light and our results indicate that CrFzl promotes the fusion of thylakoids during mating. These results suggest that membrane fusion is an essential tool for energy production in stress conditions by living organisms.

## Introduction

Dynamin-Related-Proteins (DRPs) are large GTPases that bind lipid bilayers and auto-oligomerize into high order macro-molecular structures [1]. These features provide DRPs with a dedicated ability to remodel intracellular membranes through fusion, fission and tubulation processes. The founding member of the DRP family, Dynamin 1 (Dyn1), is critical for clathrin-coated vesicle endocytosis in mammals [2]. DRPs are on the other hand essential for mitochondrial dynamics in fungi, metazoans and plants where mitochondria organize into tubular cellular networks through balanced events of division and fusion of their tubules [3,4]. In line with their strong involvement in mitochondrial dynamics and with the endosymbiotic origin of mitochondria, DRPs have evolved from prokaryotic ancestors and have been characterized in bacterial models [5,6,7]. They are highly conserved in the cyanobacterial subgroup [8] and consistent with the cyanobacterial origin of the chloroplast [9], DRPs also participate in the membrane dynamics of this fundamental photosynthetic organelle [10].

From a molecular standpoint, GTP-binding promotes Dyn1 recruitment and self-assembly into spirals around the neck of budding vesicles. GTP hydrolysis then induces constriction of the spiral resulting in scission of the lipid tube, which releases the vesicle from the plasma membrane [11]. DRP1, in mammals, and Dnm1, in yeast, act similarly to Dyn1 to promote the separation of mitochondrial tubules [1]. These “fission DRPs” are cytosolic GTPases that are recruited to their cognate membranes by specific protein and lipid adaptors. In contrast, “fusion DRPs” are generally trans-membrane GTPases [1]. Consistent with this, fusion of mitochondria is mediated by two distinct sets of DRPs that are integral to outer and inner membranes. The mitofusins (MFNs in mammals, Fzo1 in yeast) oligomerize in *cis* (*i*.*e*. on the same membrane) and then in *trans* (*i*.*e*. from opposing membranes) to tether mitochondrial outer membranes and trigger their homotypic fusion. Tethering and fusion of inner membranes is subsequently mediated by OPA1 in mammals and Mgm1 in yeast. GTP binding and hydrolysis are crucial for both membrane fusion processes, which are themselves essential for oxidative phosphorylation.

The cyanobacterial BDLP (Bacterial Dynamin Like Protein) from *Nostoc punctiforme* shares significant homology with mitofusins, suggesting its involvement in membrane fusion processes [12]. Notably, NpBDLP is soluble and self-associates as a dimer in its nucleotide-free form [12]. GTP binding promotes an extensive conformational switch of the protein from a compact structure to an open configuration [13]. In this GTP-bound state, NpBDLP inserts into lipid bilayers through a hydrophobic paddle and is able to generate tubulation of liposomes through its auto-oligomerization. *In vivo*, NpBDLP was mostly observed at the cellular internal periphery and was proposed to regulate thylakoid organization because of the cyanobacterial origin of plant chloroplasts [12].

Plant DRPs have been mainly studied in the *Arabidopsis thaliana* model, which revealed their involvement in endocytosis [14,15], in division of mitochondria and peroxisomes [16], and in cytokinesis [17]. Most importantly, plants host the essential photosynthetic electron transport chain within the highly complex and dynamic thylakoid membrane network [18]. Thylakoids reside in the chloroplast which is itself delimited by outer and inner envelope membranes. DRP5B, formerly known as ARC5, is responsible for the chloroplast division through a mechanism similar to mitochondrial fission [19]. The only other DRP known to be implicated in maintenance of the chloroplast morphology is called Fzl due to its similarity with the yeast mitofusin Fzo1 [20].

Fzl is the sole mitofusin-like protein in *Arabidopsis thaliana*. It does not regulate mitochondrial dynamics but was found inserted in the inner envelope of the chloroplast and within thylakoid membranes [20]. Its homology with NpBDLP, suggested a conserved function from cyanobacteria to plants [12]. The absence of Fzl induces a slow growth phenotype associated with the development of pale green leaves in the *Columbia* (Col-0) ecotype [20,21]. In the *Landsberg erecta* (L*er*) background, the slow growth was conserved but the older leaves developed chlorotic areas instead of homogenous bleaching [22,23]. *fzl* cells display enlarged chloroplasts with morphologically altered thylakoids. In addition, an accumulation of stromal vesicles was detected in Col-0 [20] whereas autophagosomes were visualized in the cytoplasm of fzl-L*er* mutant cells [23]. This upregulation of autophagy was associated with increased hydrogen peroxide deposit in the cell-wall and with activation of cell death signaling pathways [22,23]. The analysis of photosynthetic electron transport chain components from thylakoids of the fzl Col-0 background revealed reduced levels of chlorophyll and decreased accumulation of cytochrome *b*_*6*_*f* complex in older pale green leaves exclusively [21].

Significant but heterogeneous phenotypes have thus been linked to the absence of Fzl in Col-0 and L*er* ecotypes of *Arabidopsis thaliana*. In this context, reciprocal causality between the structural alterations of thylakoids, the photosynthetic defect observed in older leaves or the activation of autophagy and cell death pathways can hardly get established. Importantly, a possible involvement of Fzl in fusion of thylakoids has been proposed based on its similarity with mitofusins [20]. Consistent with this, cryo-electron tomography on germinating *Arabidopsis thaliana* cotyledons recently reported a delayed interconnection between developing thylakoid stacks in the absence of the DRP. Moreover, immunogold labeling localized Fzl at the points of interconnection between thylakoids [24]. Nonetheless, the established involvement of Fzl in fusion of thylakoids remains to be demonstrated and besides its potential role in architecture maintenance, the physiological function of thylakoids fusion is unknown.

With the objective of clarifying the function of Fzl in chloroplast biology and given its conservation in the green lineage [20], we focused on the counterpart of *Arabidopsis thaliana* Fzl from the unicellular microalga *Chlamydomonas reinhardtii*, which contains a single chloroplast [25]. Chlamydomonas Fzl (CrFzl) was found targeted to the chloroplast, in which it is soluble. Insertional knock out mutagenesis by CRISPR Cas9 allowed the generation of strains lacking CrFzl which revealed a requirement of the DRP during high light stress. High light treatment in the mutant induces morphological changes in the chloroplast accompanied by a significant photoinhibition of the photosynthetic electron transport chain, an accumulation of cytosolic vesicles in contact with damaged plastids and an activation of autophagy. Our observations indicate that the GTPase and hydrophobic domains of CrFzl are essential for its recruitment to intra-plastidial membranes under high light and demonstrate that CrFzl is essential for effective fusion of thylakoids during mating. Together, these results indicate that CrFzl promotes the merging of thylakoids and is required for resistance to light stress.

## Results

### CrFzl is targeted to the chloroplast

**Fig 1:**
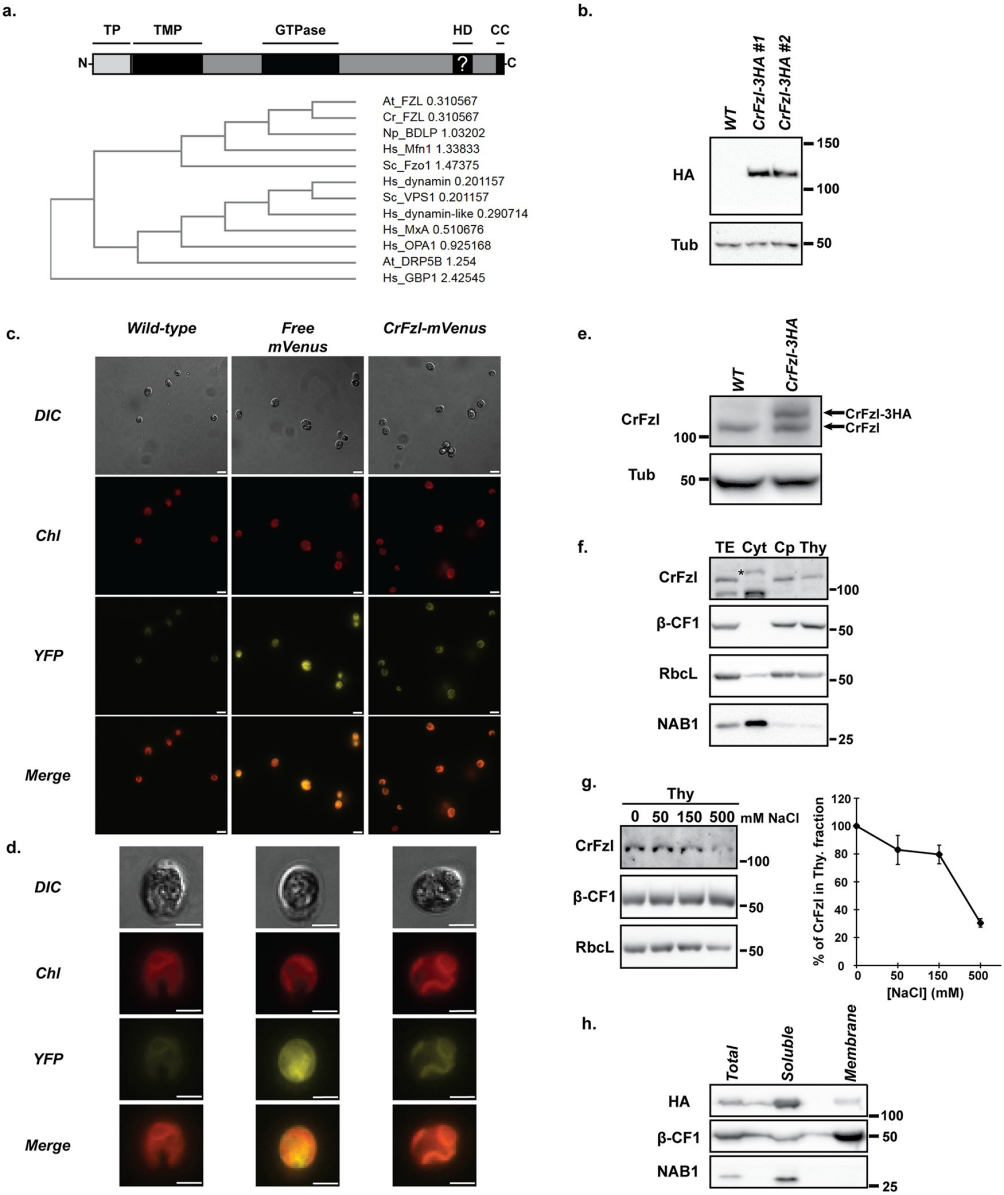
(a) *Top*. Subdomain organization of CrFzl. TP: Transit Peptide. TMP: Thiamine Mono-Phosphate synthase. HD: Potential (?) hydrophobic domain. CC: Coiled coil domain. *Bottom*. Phylogenetic tree of DRPs. GTPase domains of different DRPs were compared using the ClustalW online software. (b) Western Blot analysis of total cell extracts from wild-type and two distinct clones expressing transgenic 3HA-tagged CrFzl (CrFzl-HA #1 and #2) using HA and Tubulin antibodies. (c) Fluorescence microscopy of wild-type, free mVenus and CrFzl-mVenus expressing strains. Chlorophyll autofluorescence (Chl) is used as a chloroplast marker. Scale bar: 10 μm. (d) Close-up of single cells of wild-type, free mVenus and CrFzl-mVenus expressing strains. Scale bar: 5 μm. Representative images of phenotypes observed in 100% of the cells analyzed. (e) Western Blot analysis of total cell extracts from a wild-type and a CrFzl-3HA expressing strains using CrFzl and Tubulin antibodies. (f) Subcellular fractionation of chloroplasts and thylakoids from a wild-type strain. Thylakoid β-CF1 subunit of the ATPase complex (β-CF1), the large subunit of the RuBisCo (RbcL) and Nucleic Acid Binding protein (NAB1) were used as marker antibodies for thylakoid (Thy), chloroplast (Cp) and cytosol (Cyt) proteins, respectively. Loadings were adapted to reach similar amounts of β-CF1. The upper band appearing in the cytosolic fraction (asterisk) also appears in total extracts at higher exposure (See asterisk in S1b Fig). (g) *Left*. Western Blot analysis of the thylakoid fraction after treatment with 0, 50, 150 and 500 mM NaCl. Loadings were adapted to reach similar amounts of β-CF1. RbcL is used as a control for stromal contamination. *Right*. Quantification of CrFzl signal in the treated thylakoid fraction normalized with thylakoid marker β-CF1. Error bars represent the s.e.m. from four independent experiments. (h) Western Blot analysis of soluble and membrane fractions from a CrFzl-HA expressing strain. Thylakoid β-CF1 and NAB1 are used as membrane and soluble protein markers, respectively.

Using the Phytozome plant genome database, we found that the Cre14.g616600 locus of *Chlamydomonas reinhardtii* encodes a putative CrFzl ortholog of AtFzl. Bioinformatics analysis of the putative CrFzl protein identified a predicted GTPase domain (Fig 1a, top). The GTPase domains of CrFzl, AtFzl and NpBDLP counterparts, presented the strongest similarities with those of mitofusins (Fig 1a, bottom). Online prediction tools also identified a chloroplast transit peptide (TP), a thiamine monophosphate synthase (TMP) domain, a hydrophobic domain and one C-terminal coiled-coil fragment (Fig 1a, top). The precise properties of the hydrophobic region were uncertain as bioinformatics tools predicted two, one or no transmembrane domain at all in this region (see Materials and methods).

The genomic *CrFzl* locus was subcloned under the promoter of the PhotoSystem I (PSI) subunit PsaD and in frame with three C-terminal HA epitopes to allow constitutive expression of CrFzl-3HA in a wild-type strain. A specific signal migrating around 110 kDa was detected close to the expected molecular weight of CrFzl-3HA (CrFzl = 108 kDa + 3HA = 4.5 kDa), which validates the gene model and predicted size of CrFzl (Fig 1b). Subcellular fractionation designed to distinguish cytosolic, chloroplast and thylakoid fractions [26], revealed that CrFzl-3HA is mainly associated with the chloroplast fraction defined by the presence of the large Rubisco subunit (RbcL) and the thylakoid β-CF1 subunit of ATPase (S1 Fig). Moreover, CrFzl fused at its C-terminus with the Yellow Fluorescent Protein variant mVenus (CrFzl-mVenus) co-localized with the chlorophyll autofluorescence of the chloroplast, whereas free mVenus localized in the cytosol of *Chlamydomonas reinhardtii* cells (Fig 1c and 1d).

While these results confirm that constitutively expressed CrFzl is localized in the chloroplast, we generated polyclonal antibodies to evaluate the expression and localization of the endogenous CrFzl protein. As expected, these antibodies (anti-CrFzl) recognized both the endogenous and the 3HA-tagged CrFzl proteins (Fig 1e). In subcellular fractionation assays, endogenous CrFzl was largely associated with the chloroplast fraction positive for RbcL and β-CF1 (Fig 1f, Cp). CrFzl was also detected in the thylakoid fraction enriched for β-CF1 but this fraction was often contaminated with significant amounts of soluble RbcL (Fig 1f, Thy). Treating this thylakoid fraction with increasing amounts of salts (50, 150 and 500 mM NaCl) did not displace β-CF1, which is tightly bound to membranes, but induced progressive dissociation of both RbcL and CrFzl (Fig 1g). This suggests that CrFzl is mainly soluble in the chloroplast, which led to monitor the association of CrFzl-3HA with membranes by dissociating soluble and membrane fractions from whole cells. Interestingly, the DRP accumulated in the same fraction as the soluble Nucleic Acid Binding protein 1 (NAB1) while a smaller portion remained associated with the membrane fraction that contains β-CF1 (Fig 1h).

These results thus indicate that CrFzl is targeted to the chloroplast where the protein is mainly soluble.

### CRISPR/Cas9-engineered CrFzl mutants are sensitive to high light

**Fig 2:**
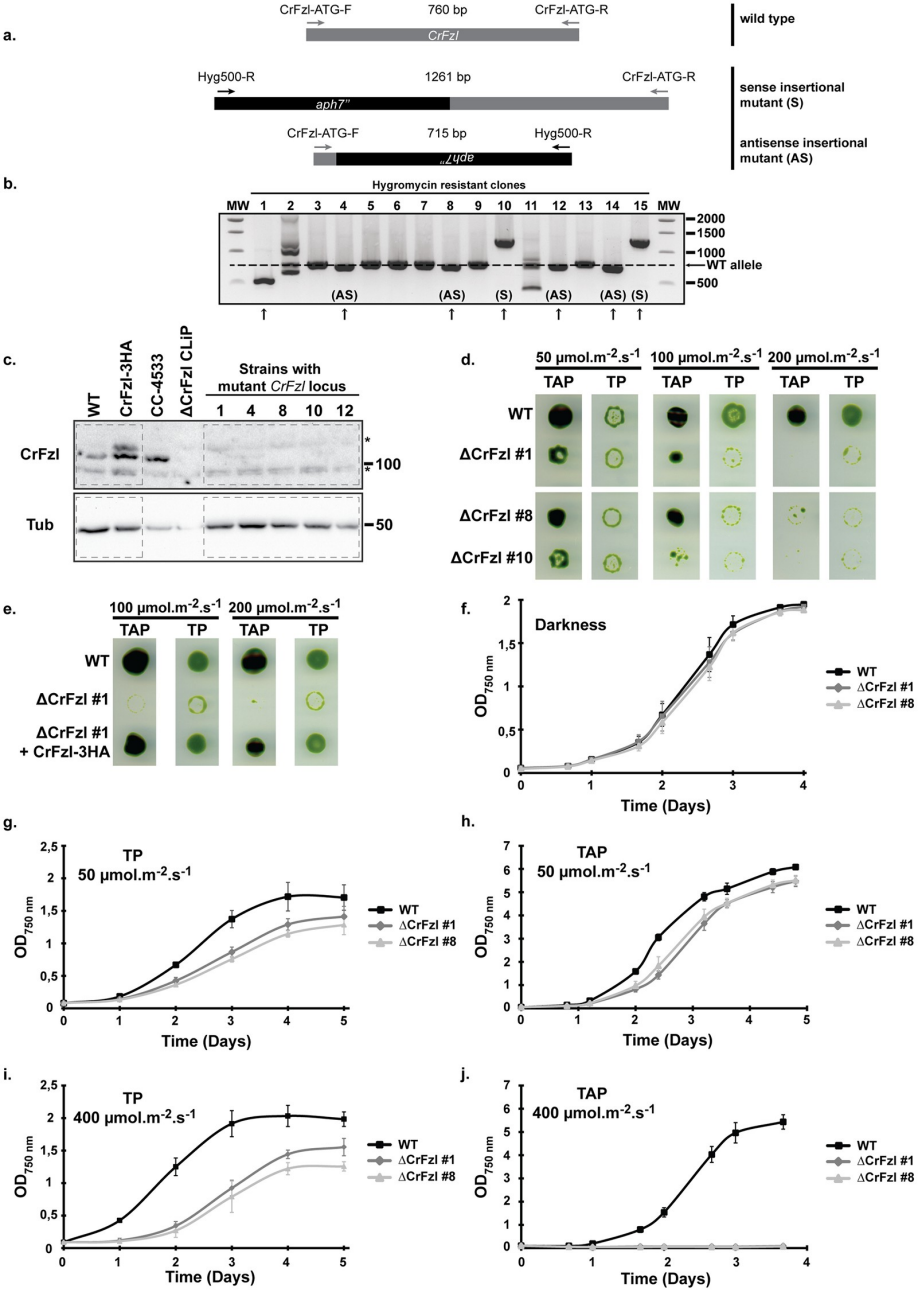
(a) Scheme of possible PCR products expected for the 3 primers PCR screen. Wild-type product is amplified after hybridization of *CrFzl* specific primers CrFzl-ATG-F and CrFzl-ATG-R, and its size is 760 bp. Expected mutant alleles hybridize with the cassette specific primer Hyg500-R and one *CrFzl* specific primer, CrFzl-ATG-F or CrFzl-ATG-R, allowing the amplification of a product of 715 bp or 1261 bp, respectively. (b) PCR products obtained for the first fifteen hygromycin resistant strains. Dashed line represents the wild-type band size. Arrows highlight strains displaying a non wild-type band mainly corresponding to a sense (S) or an antisense (AS) insertion. In the clone #1, an additional deletion of187 bp occurred during integration of the cassette, without affecting the functionality of the cassette as the full RBCS2 terminator sequence remains (see S2c Fig). (c) Western Blot analysis with α-CrFzl and α-Tubulin antibodies of the first five hygromycin resistant strains displaying a non-wild-type PCR product. Wild-type and CrFzl-3HA are used as positive control of the same genetic background as the mutant strains. LMJ.RY0402.175738 (ΔCrFzl CLiP) from the CC-4533 background is used as knock out control. Asterisks correspond to non-specific signals. (d) Spot assay of the parental wild-type and 3 ΔCrFzl strains on rich (TAP) and minimum (TP) media exposed to 50, 100 or 200 μmol.m^-2^.s^-1^ light irradiance. (e) Spot assay of the parental wild-type, the ΔCrFzl strain #1 and the strain complemented with CrFzl-3HA in the same media as in (d) and exposed to 100 and 200 μmol.m^-2^.s^-1^. (f) Liquid growth curve of the parental wild-type and 2 ΔCrFzl strains in heterotrophic conditions (TAP + darkness). (g) Liquid growth curve of the parental wild-type and 2 ΔCrFzl strains in photoautotrophic conditions (TP) and moderate light (50 μmol.m^-2^.s^-1^). (h) Liquid growth curve of the parental wild-type and 2 ΔCrFzl strains in mixotrophic conditions (TAP) and moderate light (50 μmol.m^-2^.s^-1^). (i) Liquid growth curve of the parental wild-type and 2 ΔCrFzl strains in photoautotrophic conditions (TP) and high light (400 μmol.m^-2^.s^-1^). (j) Liquid growth curve of the parental wild-type and 2 ΔCrFzl strains in mixotrophic conditions (TAP) and high light (400 μmol.m^-2^.s^-1^). (f-j) Error bars represent the s.e.m. from three independent experiments.

Functional analysis of CrFzl required strains that lack expression of the protein. The Chlamydomonas Mutant Library [27] contains one strain, LMJ.RY0402.175738, in which a paromomycin resistance cassette is inserted in the open reading frame of the Cre14.g616600 locus. However, preliminary analyzes revealed that this strain presents phenotypes that do not segregate with the insertion cassette, suggesting additional mutations in its genome. We thus took the challenge of generating CrFzl knock out strains using the still emerging CRISPR/Cas9 technology in *Chlamydomonas reinhardtii* [28,29,30,31]. We designed a small guide RNA (sgRNA) that targets a 20 bp sequence from the first exon of the Cre14.g616600 locus, leading to a cleavage by the Cas9 nuclease after the 66th nucleotide (S2a Fig). The wild-type cw15.J3 strain was co-transformed with the sgRNA/Cas9 RNP complex and a cassette containing the AphVII hygromycin resistance gene. Roughly 800 hygromycin resistant colonies were obtained and 322 of those were screened using a PCR strategy with 3 distinct primers (S2b Fig) expected to generate 3 distinct products (Fig 2a): a 760 bp PCR product when the CrFzl locus is uninterrupted and 1261 bp (sense) or 715 bp (antisense) products depending on the orientation of the cassette upon insertion at the expected genomic site. Out of 322 candidate strains subjected to PCR screening, 153 (47.5%) displayed a product different from the 760 bp wild-type PCR fragment (Fig 2b; the screen for the first 15 clones is shown). Among these, 41 presented the sense product (Fig 2b, S, clones #10 and #15), 62 the antisense fragment (Fig 2b, AS; clones #4, #8, #12 and #14) and 48 clones had more complex rearrangements resulting in different lengths and number of the PCR products (Fig 2b, clone #1, #2 and #11). We sequenced the flanking regions of the predicted cut site at the CrFzl locus of 4 distinct clones (#1, #8, #10 and #12) and confirmed insertion of the resistance cassette at that site (S2c Fig).

Expression of the endogenous protein in five candidate CrFzl mutants (clones #1, #4, #8, #10 and #12) was then assessed by western blot of total cellular extracts with the anti-CrFzl (Fig 2c, dashed boxes). The wild-type strain from the Chlamydomonas Mutant Library, CC-4533, and the mutant strain LMJ.RY0402.175738 (ΔCrFzl CLiP) for which the cassette insertion is predicted in the 8th intron were used as additional controls. We confirmed the successful CRISPR/Cas9-based inactivation of CrFzl since its expression was undetectable in all mutants. The CRISPR/Cas9 technology thus allowed the generation of several strains defective for the expression of CrFzl in a timely fashion (6 days for selection of hygromycin resistant strains, 4 days for amplification of isolated mutants, 1 day for DNA extraction and PCR and 3 days for growth, protein extraction and western blotting of selected mutants).

The effect of abrogating CrFzl expression was first assessed by growth of wild-type and CrFzl mutant strains in mixotrophy on TAP medium (growth relies on supplied acetate and photosynthesis) or in photoautotrophy on minimal medium (TP, growth solely relies on photosynthesis). On TAP medium, three distinct CrFzl mutants (clones #1, #8 and #10) displayed a slight growth defect as compared to the wild-type (Fig 2d, TAP, 50 μmol photons.m^-2^.s^-1^). On minimal medium, the wild-type strain grew better with higher light intensity (compare 50, 100 and 200 μmol photons.m^-2^.s^-1^), whereas growth of the mutants was nearly abrogated at 100 and 200 μmol photons.m^-2^.s^-1^ (Fig 2d). In mixotrophic conditions, the growth of mutant strains was also gradually impaired with higher irradiance (Fig 2d, TAP, 100 μmol photons.m^-2^.s^-1^, 200 μmol photons.m^-2^.s^-1^). Importantly, reintroducing CrFzl expression in the mutants fully rescued the growth phenotype, both on TP and TAP media under all light regimes (Fig 2e). These results thus demonstrate that CrFzl is required to maintain cell division and growth under high light.

Consistent with this observation, the growth of mutant strains (clones #1 and #8) was undistinguishable from that of the wild-type in heterotrophic conditions, *i*.*e*. liquid TAP medium and darkness (Fig 2f). In contrast, moderate light (50 μmol photons.m^-2^.s^-1^) induced a slight delay in growth induction of the mutants in both TAP and TP liquid media (Fig 2g and 2h). Most strikingly, growth of the mutants in high light regime (400 μmol photons.m^-2^.s^-1^) was strongly delayed in TP and even completely inhibited in TAP liquid cultures (Fig 2i and 2j).

CRISPR/Cas9 mutagenesis thus allowed the successful generation of multiple ΔCrFzl mutants and their preliminary analysis revealed the requirement of CrFzl for survival in conditions of high illumination.

### CrFzl is required for resistance against photoinhibition

**Fig 3:**
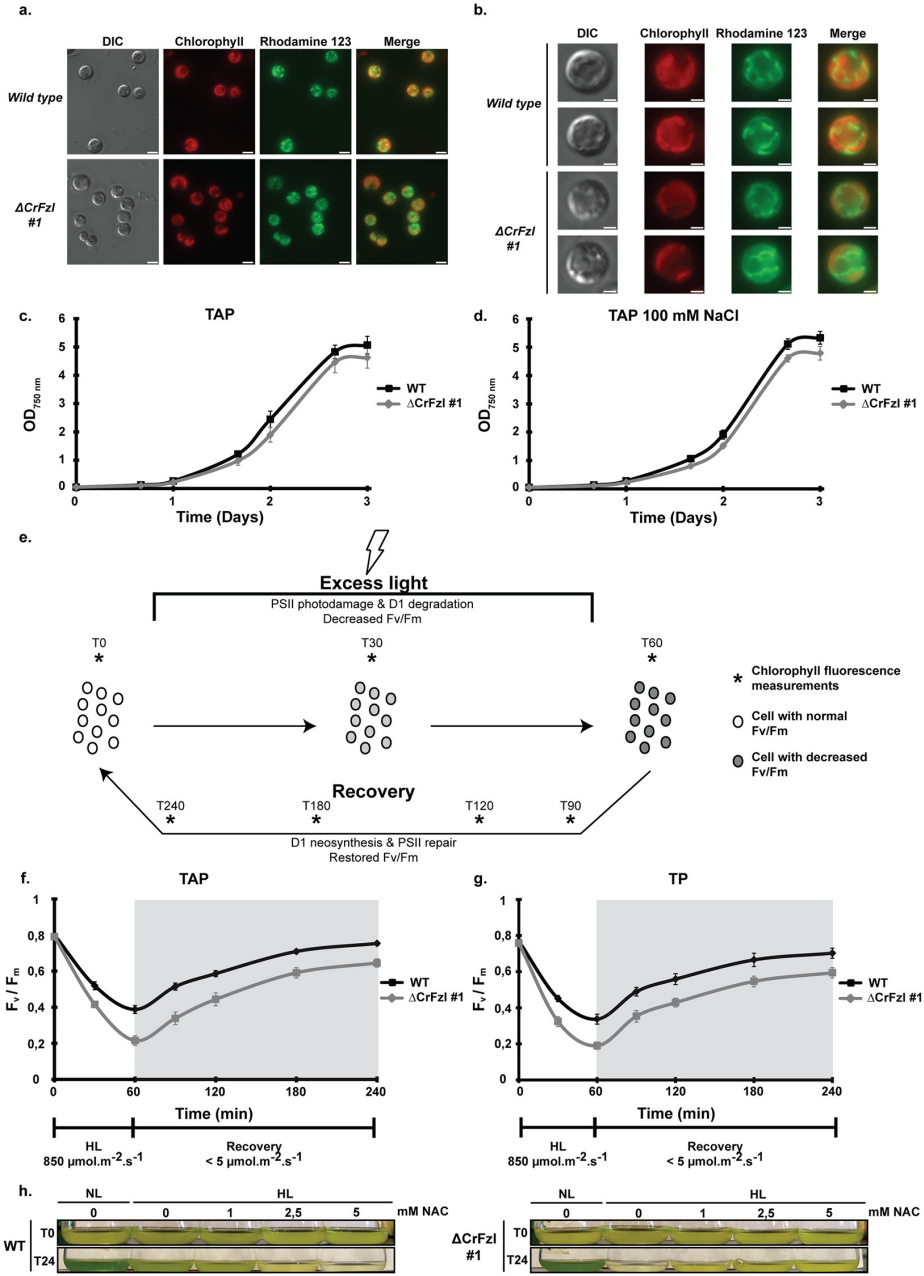
(a) Fluorescence microscopy images of wild-type and ΔCrFzl #1 strains stained with the mitochondrial dye Rhodamine 123. Chlorophyll autofluorescence is used as a chloroplast marker. Scale bar: 5 μm. (b) Close-up of single cells from the Rhodamine 123 stained wild-type and ΔCrFzl #1 strains. Scale bar: 2 μm. (c) Liquid growth curve of the wild-type and ΔCrFzl #1 strains in control mixotrophic, normal light conditions. Error bars represent the s.e.m. from three independent experiments. (d) Liquid growth curve of the wild-type and ΔCrFzl #1 strains in mixotrophic, normal light conditions with high salt (100 mM) concentration. Error bars represent the s.e.m. from three independent experiments. (e) Principle of the photoinhibition experiment. Cell cultures are subjected to excess light stress for 1 hour leading to overall decrease of Fv/Fm in the cell population due to PSII photo-damage and degradation of the D1 subunit. Incubation in very low light allows for recovery of full photosynthetic capacity after 3 hours. Asterisks correspond to time-course measurements. Error bars represent the s.e.m. from three independent experiments. (f) Time course of photoinhibition at 850 μmol.m^-2^.s^-1^ (HL) and recovery at very low light of the wild-type and the ΔCrFzl #1 strain in TAP medium, monitored by PSII quantum yield (F_v_/F_m_). (g) Time course of photoinhibition at 850 μmol.m^-2^.s^-1^ (HL) and recovery at very low light of the wild-type and the ΔCrFzl #1 strain in minimal TP medium, monitored by PSII quantum yield (F_v_/F_m_). Error bars represent the s.e.m. from three independent experiments. (h) Antioxidant rescue of high light induced cell death. Cells of wild type and the ΔCrFzl #1 mutant background were diluted to a OD_750nm_ ~1 (T0), supplemented with N-acetyl-cysteine as indicated and grown in normal (NL) or high (HL) light conditions for 24 hours.

Growth on agar and in liquid media indicated a high sensitivity of ΔCrFzl mutant strains to light stress. Consistent with a specific effect on the chloroplast, we observed that both wild-type and mutant strains display undistinguishable tubular mitochondrial networks when stained with the mitochondrial dye rhodamine 123 (Fig 3a and 3b). This confirms that, in contrast to its yeast homolog Fzo1, Chlamydomonas Fzl is not involved in the regulation of mitochondrial dynamics [20]. Moreover, the wild-type strain and a CrFzl mutant were grown under normal light (50 μmol photons.m^-2^.sec^-1^) in TAP medium with or without NaCl to assess the effect of high salt concentration, another abiotic stress [32], upon loss of CrFzl expression. As expected, incubation under normal light in TAP medium without NaCl caused a very weak delay in the induction of growth for the mutant strains (Fig 3c). However, increasing salt concentration to 100 mM NaCl [33] did not further affect growth of the ΔCrFzl strain (Fig 3d), confirming that the growth phenotypes we observed are not affected by high salt and may thus be specifically associated with light stress.

During exposure to high light, photosynthetic electron transport is saturated and chlorophylls excited by light will dissipate their energy by forming reactive oxygen species (ROS), inducing damage to the photosynthetic machinery. Excess damage promotes degradation of the D1 protein, which is the core subunit of Photosystem II (PSII), resulting in partial inactivation of the global PSII pool and photoinhibition [34]. This manifests as a decrease of the maximum PSII quantum yield (Fv/Fm, see Material and methods). This indicator of PSII efficiency can be monitored *in vivo* by measurements of chlorophyll fluorescence during photoinhibition (Fig 3e). The wild-type strain and a ΔCrFzl mutant, grown in TAP or TP medium, were thus exposed to photoinhibitory light irradiance (850 μmol photons.m^-2^.s^-1^) during one hour followed by recovery in very low light for three hours. In both growth conditions, a significant decrease of the PSII quantum yield was observed in the ΔCrFzl mutant upon photoinhibition whereas both strains recovered from light induced damage at a similar rate (Fig 3f and 3g), These results therefore indicate that CrFzl protects photosynthesis against photoinhibition. This protection may allow limiting the amount of ROS induced by photoinhibition, whereas excess ROS may contribute to the growth inhibition in high light of ΔCrFzl mutants (Fig 2j). In agreement with these possibilities, the growth of the wild-type strain was progressively inhibited with increasing concentrations of NAC (Fig 3h; WT) whereas the ΔCrFzl mutant was progressively rescued by the antioxidant (Fig 3h; ΔCrFzl). CrFzl is thus required for growth during high light stress to possibly protect photosynthesis against ROS generated during photoinhibition.

### CrFzl is required for chloroplast integrity under high light

Our data indicate that CrFzl is required for growth during light stress and that strong irradiance induces reduced resistance to photoinhibition in the mutant. Given that CrFzl belongs to the DRP membrane remodeling factors family, we hypothesized that these defects might result from perturbations in the plastid membrane system. We thus examined the morphology of the chloroplast taking advantage of the fluorescence emitted by the chlorophyll pigment that is mainly inserted in the thylakoid membrane.

**Fig 4:**
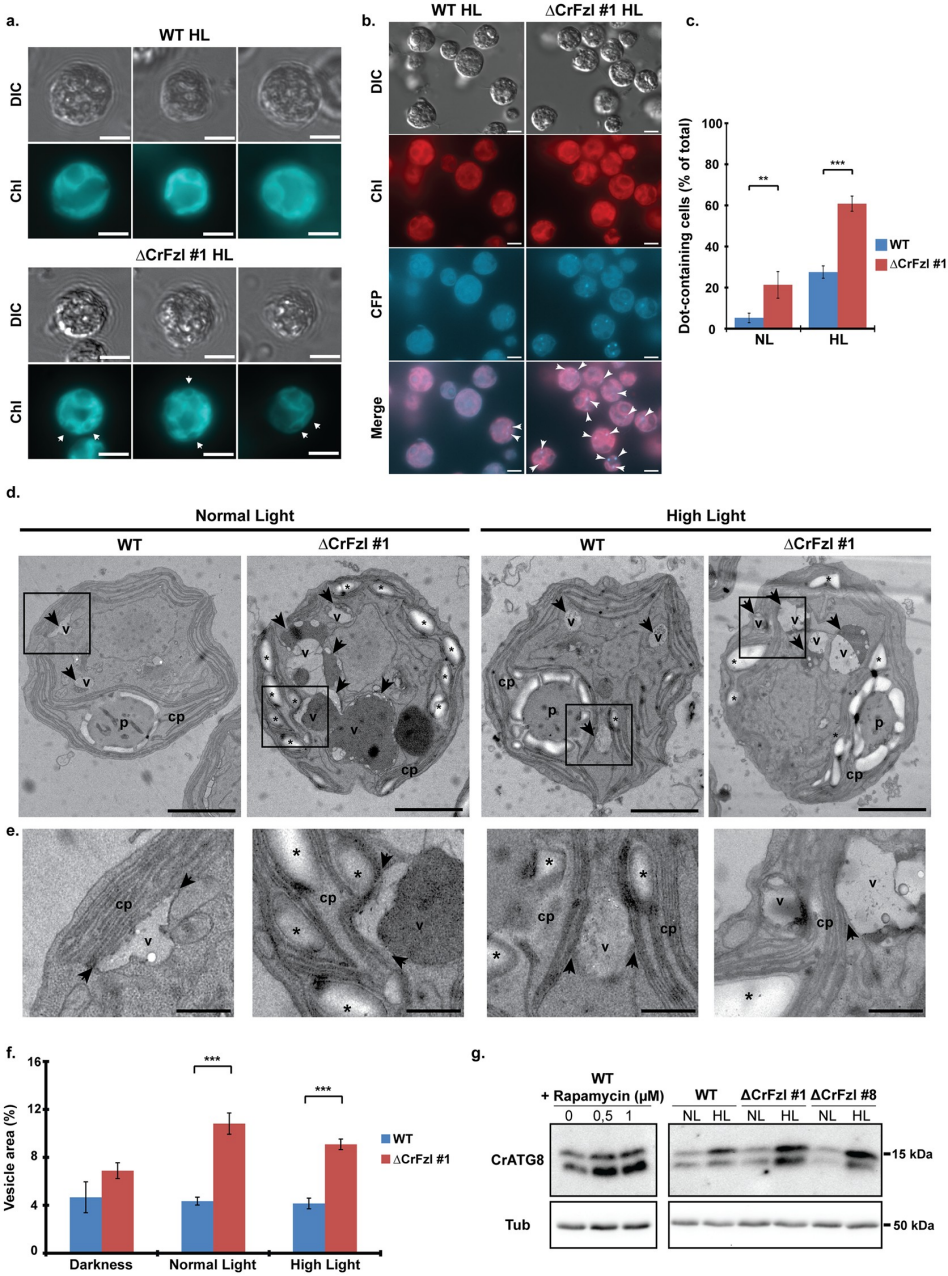
(a) Fluorescence microscopy of wild-type and ΔCrFzl #1 chloroplasts after 6 hours of light stress. Chlorophyll autofluorescence (Chl) is used to analyze chloroplast morphology. Scale bar: 5 μm. Arrows indicate dark structures within the fluorescent lobes of the plastids. (b) Fluorescence microscopy of wild-type and ΔCrFzl #1 cells after 6 hours of light stress. Images were taken with wavelength/filter sets used for detection of CFP and chlorophyll (Chl). Scale bar: 5 μm. Arrowheads indicate auto-fluorescent dots detected with the CFP filter set. (c) Percentage of cells with fluorescent dots after incubation in normal (NL) or high light (HL) during 6 hours. Error bars represent the s.d. from five independent experiments. Total number of cells analyzed, WT NL: 1227; WT HL: 1869; ΔCrFzl NL: 1284; ΔCrFzl HL: 1789. ** p < 0,05. *** p < 0,005 (Student test). (d) Electron micrographs of wild-type and ΔCrFzl #1 cells exposed to normal and high light. cp: chloroplast; p: pyrenoid; arrows/v: vesicles; asterisks: starch granules. Scale bar: 2 μm. Squares indicate the close up regions in (e). (e) Close up region of vesicle-chloroplast contact regions in cells from (d). cp: chloroplast; v: vesicles; asterisks: starch granules; arrows: zone of contact between vesicle and plastid envelope. Scale bar: 500 nm. (f) Percentage of the total cellular area occupied by vesicles in wild-type and ΔCrFzl #1 cells in darkness, normal and high light conditions. Error bars represent the s.e.m. from the cellular area occupied by vesicles in the same number of cells analyzed in WT Darkness: 71; WT NL: 91; WT HL: 56; ΔCrFzl Darkness: 93; ΔCrFzl NL: 88; ΔCrFzl HL: 69. ** p < 0,05. *** p < 0,005 (Student test). (g) Western blot analysis of ATG8 induction in response to high light treatment. *Left*. As a control of ATG8 induction, the wild type strain was treated with 0,5 and 1 μM Rapamycin for 16 hours. *Right*. Cells from wild type and mutant (#1 and #8) background were treated with high light for 6 hours before extraction of the proteins.

Under low irradiance, the plastids from wild-type and CrFzl mutant strains are detected as 2 lobes appressed against the plasma membrane and joining around the pyrenoid where chlorophyll fluorescence cannot be seen as it mostly consists in non-chlorophyll-associated proteins [35,36] (Fig 4a and S3a Fig). After 6 hours of light stress, this typical cup shape of the chloroplast was unchanged in the wild-type strain but ΔCrFzl cells accumulated dark structures within the fluorescent lobes of the plastids (Fig 4a, arrows).

While analyzing the cells with distinct wavelengths and filter sets, we also detected fluorescent dot-like structures (Fig 4b, S3b Fig, arrowheads). These dots were spotted with settings usually used for detection of DAPI, CFP, GFP or YFP but not for detection of chlorophyll (Chloro setting), indicating that their spectral properties spanned wavelength of 365–505 nm for excitation and 397–600 nm for emission. In normal light conditions, the fluorescent dots were hardly detected in wild-type cells (< 3%) but seen in more than 20% of mutant cells (Fig 4c). High light stress increased their occurrence by 3-fold in both wild-type (27%) and mutant cells (63%). The origin of their fluorescence is unknown but these dots appeared nearly systematically located in the cytoplasm and in close juxtaposition with the chloroplast (Fig 4b).

Conventional transmission electron microscopy (TEM) analysis revealed that absence of CrFzl in fact induces the accumulation of starch granules in lobe regions of the chloroplast (Fig 4d and 4e and S3c Fig; asterisks). Notably, this increase in starch granules correlated with an altered shape of the pyrenoid that, in normal light conditions, was clearly identifiable in only ~7% of mutant cells against 20% in the wild-type (S3d Fig). These starch granules devoid of chlorophyll likely correspond to the dark structures that accumulate in the fluorescent lobes of plastids from ΔCrFzl cells.

Another important feature of the ΔCrFzl strain analyzed by TEM was the systematic observation of very large cytoplasmic vesicular structures in both normal and high light conditions (Fig 4d and 4e, S3 Fig; arrows and v). In the ΔCrFzl strain, these vesicles were much larger than vesicles seen in the wild-type (Fig 4f) and were filled with electron dense material (Fig 4d). Notably, the vesicles and the chloroplast were in such contact that the plastid envelope was often undistinguishable from the membrane of the vesicle (Fig 4e; arrows). This led us evaluating a possible involvement of autophagy by monitoring the expression of ATG8 which is known to be stimulated upon induction of autophagy by Rapamycin ([37] and Fig 4g). Interestingly, the levels of this autophagy marker were increased upon strong illumination of WT cells and this increase was even stronger in ΔCrFzl mutants (Fig 4g).

Upon treatment with high light, cells lacking CrFzl thus simultaneously combine two features that may relate to the degradation of the chloroplast: an activation of autophagy (Fig 4g) and large vesicles that adjoin the chloroplast and may correspond to vacuoles (Fig 4d and 4e). In this context, carotenoids that absorb light between 400 and 520nm [38] and that accumulate in thylakoid membranes may represent candidates for the fluorescence of the spots that were found in the vicinity of the plastid (Fig 4b and 4c).

These results indicate that lack of CrFzl perturbs the organization of the chloroplast. This may be linked to the decreased resistance to photoinhibition during light stress, which would lead to degradation of the plastid and cell death upon extended exposure. This prompted us to begin investigating the mean by which CrFzl regulates the morphology of the chloroplast.

### CrFzl is recruited to intra-plastidial membranes upon high illumination

**Fig 5:**
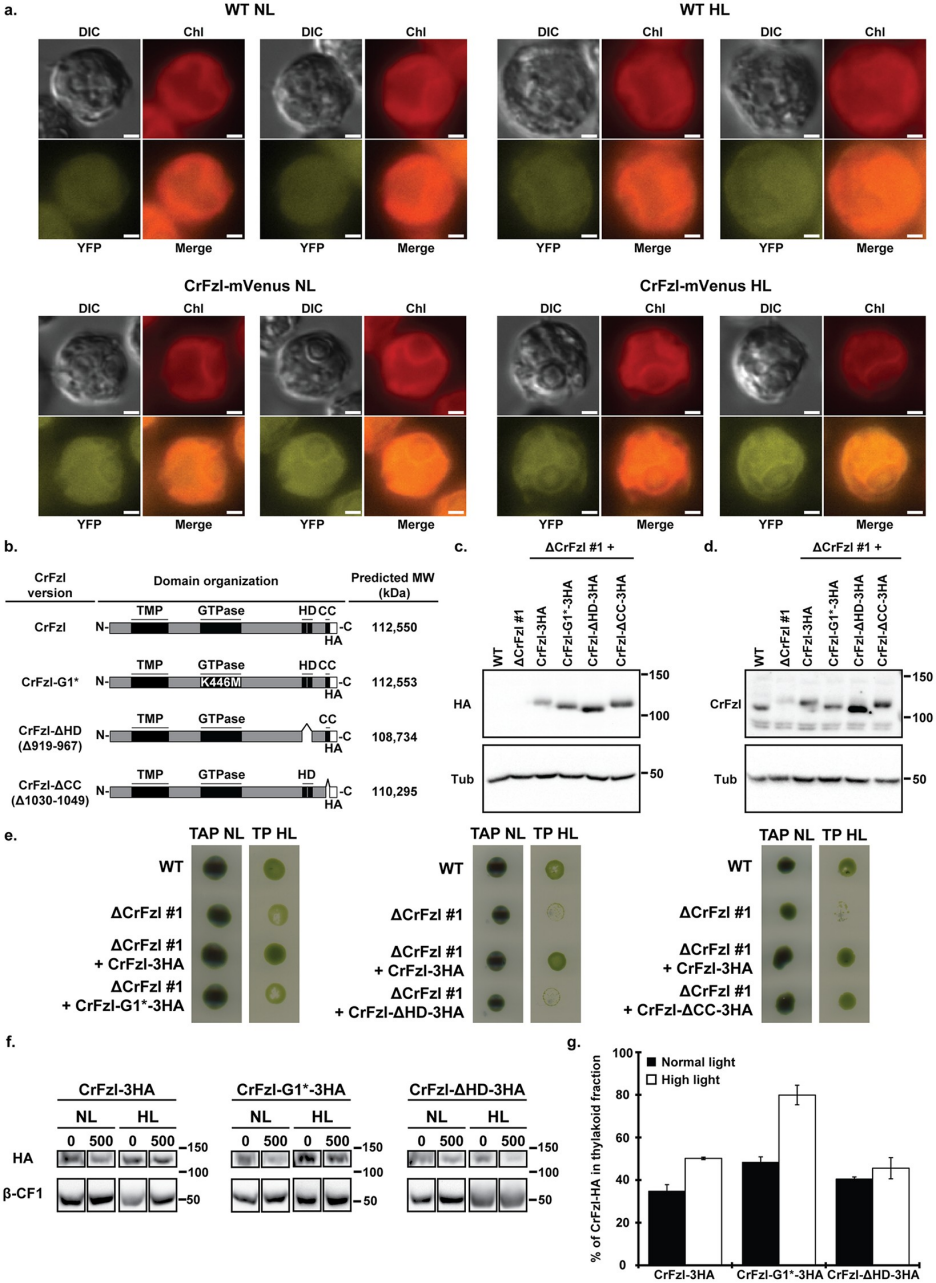
(a) Fluorescence microscopy of cells from wild-type (top) and a ΔCrFzl strain expressing the CrFzl-mVenus construct (bottom) after growth in normal (NL) and high light (HL) conditions. Chlorophyll autofluorescence is used as a thylakoid membranes marker. Scale bar: 1μm. (b) Subdomain organization of the distinct CrFzl-3HA versions expressed in the ΔCrFzl mutant background. (c-d) Western blot analysis of the expression of the distinct CrFzl-3HA versions expressed in the ΔCrFzl mutant with anti-Tubulin, anti-HA (c) and anti-CrFzl (d). Untransformed wild-type and ΔCrFzl strains are used as controls. (e) Spot assay of the parental wild-type, the ΔCrFzl strain #1, the strain ΔCrFzl #1 complemented with CrFzl-3HA and ΔCrFzl #1 expressing a mutant version of CrFzl-3HA (left panel: CrFzl-G1*-3HA; middle panel: CrFzl-ΔHD-3HA; right panel: CrFzl-ΔCC-3HA) on TAP at normal light (NL, 50 μmol.m^-2^.s^-1^) as a control, and on minimal TP medium at 100 μmol.m^-2^.s^-1^ (TP HL). (f) Western blot analysis of CrFzl-3HA, CrFzl-G1*-3HA and CrFzl-ΔHD-3HA in thylakoid fractions washed with 0 and 500 mM NaCl after 6 hours of normal (NL) or high light (HL) treatment. Loadings were adapted to reach equivalent amounts of β-CF1. 0 and 500 mM NaCl samples were loaded on the same gels; uncut western blots are available in S4c Fig. (g) Percentage of CrFzl-3HA, CrFzl-G1*-3HA or CrFzl-ΔHD-3HA left in thylakoid fractions washed with 500 mM NaCl after 6 hours of normal (NL) or high light (HL) treatment. HA signal was normalized to the β-CF1 signal and compared with the amount found in thylakoids washed without NaCl. Error bars represent the s.e.m. from three independent experiments.

The high light phenotypes induced by the absence of CrFzl likely result from defects that are secondary to the loss of function of the DRP. We thus reasoned that following the behavior of the CrFzl protein during high light stress could begin hinting at its primary function. For this purpose, we generated a ΔCrFzl strain expressing CrFzl-mVenus as the unique source of CrFzl in the cell and followed the distribution of the protein in normal and high light conditions by fluorescence microscopy (Fig 5a, S4a Fig). In the wild-type strain expressing the untagged CrFzl, a diffuse background signal was detected with the YFP filter set in both normal and high light conditions but this signal did not demarcate any specific subcellular structure (Fig 5a; WT NL, WT HL). In CrFzl-mVenus cells, a specific YFP signal was detected. In normal light (NL), this YFP signal delimited tubular structures and colocalized with the fluorescence of Chlorophyll, which is inserted in thylakoids membranes. Most importantly, this tubular labelling seemed stimulated under high light (HL). These results suggest that CrFzl may be recruited to thylakoid membranes and that this recruitment would be stimulated upon high light stress.

To confirm this possibility, we reasoned that the dynamic recruitment of CrFzl onto intra-plastidial tubules may require integrity of the distinct sub-domains of the DRP. We thus began by evaluating the capacity of CrFzl mutants (Fig 5b) to rescue the strong phenotypes generated by the absence of CrFzl. Wild-type CrFzl-3HA or CrFzl-3HA mutant versions with a substitution of a methionine for the conserved lysine 446 within the G1 section of the GTPase domain (CrFzl-G1*-3HA), a deletion of the predicted hydrophobic domain (CrFzl-ΔHD-3HA) or a deletion of the C-terminal coiled-coil domain (CrFzl-ΔCC-3HA) were expressed in ΔCrFzl cells (Fig 5c and 5d). The resulting strains were then monitored for their growth on complete media under low light (TAP) or minimal media under high light (TP) as compared to the WT, ΔCrFzl and CrFzl-3HA control strains (Fig 5e). While the expression of wild-type CrFzl-3HA or CrFzl-ΔCC-3HA totally corrected the growth defect of ΔCrFzl on TP media under high light, the GTPase and the ΔHD mutants lost this rescuing capacity. Notably, the same conclusions were obtained by assessing the occurrence of fluorescent dots to monitor CrFzl functionality (S4b Fig). The integrity of the GTPase domain and HD region of CrFzl are thus essential for the function of the DRP whereas the predicted C-terminal CC domain is dispensable.

These findings led us isolating the thylakoid fractions from CrFzl-3HA, CrFzl-G1*-3HA and CrFzl-ΔHD-3HA cells that were grown under normal light (NL) or under high light (HL) in TAP medium. We then quantified the amount of CrFzl remaining on each thylakoid fractions before and after washing with 500 mM NaCl (Fig 5f). Importantly, this approach revealed that the binding of WT CrFzl-3HA to thylakoids is stronger under high light (50% remaining) than under low light (35% remaining), thus confirming that the recruitment of CrFzl to thylakoids is stimulated upon strong illumination (Fig 5g; CrFzl-3HA). More surprisingly, we observed that while the amount of CrFzl-ΔHD-3HA associated with intra-plastidial membranes was not significantly modified by light (Fig 5g; CrFzl-ΔHD-3HA), that of CrFzl-G1*-3HA increased by more than 35% upon strong illumination (Fig 5g; CrFzl-G1*-3HA).

These observations are consistent with the recruitment of CrFzl on thylakoids upon high light stress. In addition, the hydrophobic and GTPase domains would regulate this recruitment to preserve the integrity of thylakoids, possibly by triggering their fusion.

### CrFzl is required for thylakoid fusion

**Fig 6:**
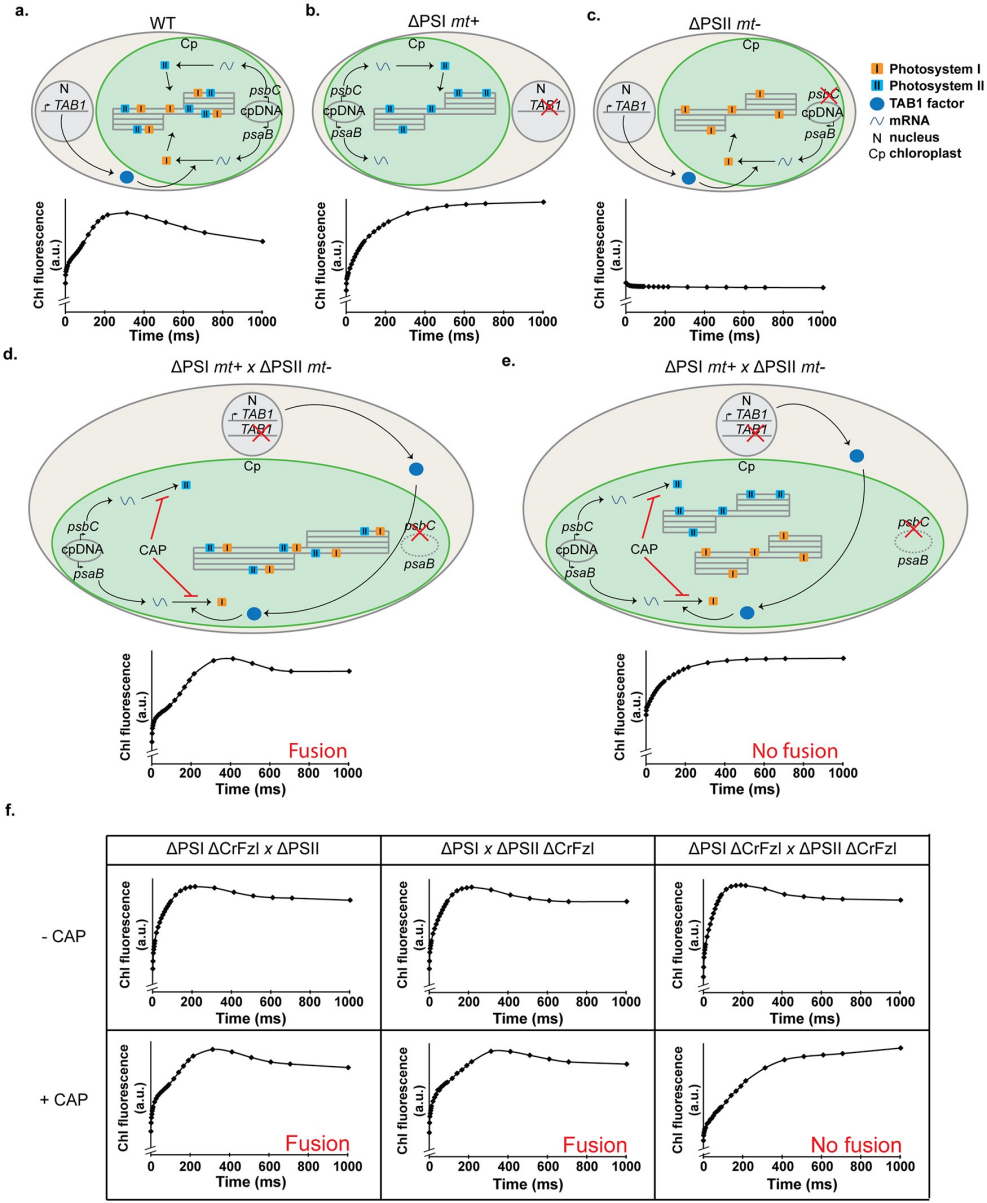
(a-c) *Top schemes*. Representation of a wild-type (a), a ΔPSI (b) and a ΔPSII (c) cell in regard to the synthesis of the subunits of interest. In a wild-type strain (a), PSI subunit is encoded by the *psaB* gene on the chloroplast genome. mRNA translation is allowed by the nuclear encoded factor TAB1. PsaB is then inserted in thylakoid membranes as part of the PSI. On the other hand, the PSII subunit PsbC is also encoded on the chloroplast genome and will be translated before insertion in the PSII. In the ΔPSI mutant F15 (b), the translation factor TAB1 is missing, preventing translation of the PsaB subunit and assembly of PSI. In the ΔPSII strain Fud34 (c), a mutation in the 5’UTR of the *psbC* mRNA induces the formation of a secondary structure that prevents its translation and assembly of the PSII. *Bottom graphs*. Chlorophyll fluorescence induction kinetic of the corresponding strains. (d) *Top scheme*. Representation of a zygote from a ΔPSI x ΔPSII cross in presence of chloramphenicol (CAP) and after thylakoid fusion. CAP prevents translation of both plastid encoded photosystem subunits PsaB and PsbC, and therefore the *de novo* assembly and insertion of functional PSI and PSII in parental thylakoid membranes. Pre-existing PSI and PSII complexes are mixed following fusion of the thylakoid membranes. Note that during mating, chloroplast DNA of the *mt-* parental strain is degraded. *Bottom graph*. Chlorophyll fluorescence induction kinetic of the zygote with fused thylakoid showing recovery of photosynthetic electron transfer witnessed by fluorescence relaxation as in (a).. (e) *Top scheme*. Representation of a zygote with non-fused thylakoids. *Bottom graph*. Chlorophyll fluorescence induction kinetic of ΔPSI and ΔPSII cells mixed in equal amount to mimic a lack of thylakoid fusion. (f) Chlorophyll fluorescence induction kinetics from zygotes in the different crosses performed in the absence or in the presence of CAP.

To directly assess the capacity of CrFzl to mediate fusion between thylakoids, we turned to the only thylakoid fusion assay established to date [39]. This assay requires two distinct strains with opposing mating types (*mt*) in which the photosynthetic activity is abolished: the *mt+* Photosystem I mutant F15 (hereafter referred as ΔPSI) and the *mt-* Photosystem II mutant Fud34 (ΔPSII). ΔPSI is a mutant of the nuclear gene *TAB1*, whose absence prevents the translation of the PSI subunit PsaB from the plastid genome (Fig 6a and 6b, top schemes) [40,41]. ΔPSII is a mutant of the plastid DNA in the 5’UTR of the gene encoding the PSII subunit PsbC, which prevents its translation (Fig 6a and 6c, top schemes) [42]. When ΔPSI is crossed with ΔPSII, the chloroplasts of each mutant fuse together and functional complementation in the zygote allows recovery of photosynthetic activity by *de novo* synthesis of PSI and PSII subunits. In the presence of chloramphenicol (CAP), *de novo* synthesis of PSI and PSII subunits is prevented and the recovery of photosynthetic activity can be achieved if and only if the chloroplasts from parental strains mix their respective contents after fusion and their thylakoids then fuse together and mix their pre-existing PSI and PSII complexes (Fig 6d, top scheme).

The photosynthetic activity in parental strains and zygotes can be assessed as previously described by measurement of chlorophyll fluorescence induction kinetics [39]. In a wild-type strain, chlorophyll fluorescence progressively rose upon continuous illumination due to PSII photoreduction, and then decreased upon reoxidation because electrons were transferred toward PSI (Fig 6a, bottom graph). In the PSIΔ strain, chlorophyll fluorescence was induced as in the wild-type but did not decrease because no PSI can receive the electrons from PSII (Fig 6b, bottom graph). In the PSIIΔ strain, chlorophyll fluorescence was low but readily saturated because emitted photons cannot get derived to PSII (Fig 6c, bottom graph). After mating of ΔPSI and ΔPSII strains in the presence of CAP, the progressive increase in fluorescence and its subsequent decrease were detected as in wild-type cells because thylakoid fusion occurred (Fig 6d, bottom graph). We also mixed ΔPSI and ΔPSII strains and took fluorescence measurements before mating to mimic a lack of thylakoid fusion (Fig 6e, top scheme). In this context, the progressive increase occurred but no subsequent decrease in fluorescence could be detected (Fig 6e, bottom graph).

After confirming that chlorophyll fluorescence induction kinetics is a reliable readout to assess photosynthesis activity and thylakoid fusion, we repeated these experiments by crossing ΔPSI and ΔPSII strains with ΔPSII ΔCrFzl and ΔPSI ΔCrFzl strains, respectively (Fig 6f, first and second columns). The crosses were performed in the presence of CAP to assess thylakoid fusion but also in its absence to monitor mating efficiency by functional complementation. Photosynthesis was rescued in each situation indicating efficient mating (-CAP) and functional homotypic fusion of chloroplast and thylakoid membranes (+CAP). In striking contrast, we observed that upon crossing of ΔPSI ΔCrFzl with ΔPSII ΔCrFzl, photosynthesis was rescued in the absence but not in the presence of CAP (Fig 6f, third column and S5 Fig). This indicates that CrFzl is not required for mating or for fusion of chloroplasts but is required for the fusion of thylakoids.

**Fig 7:**
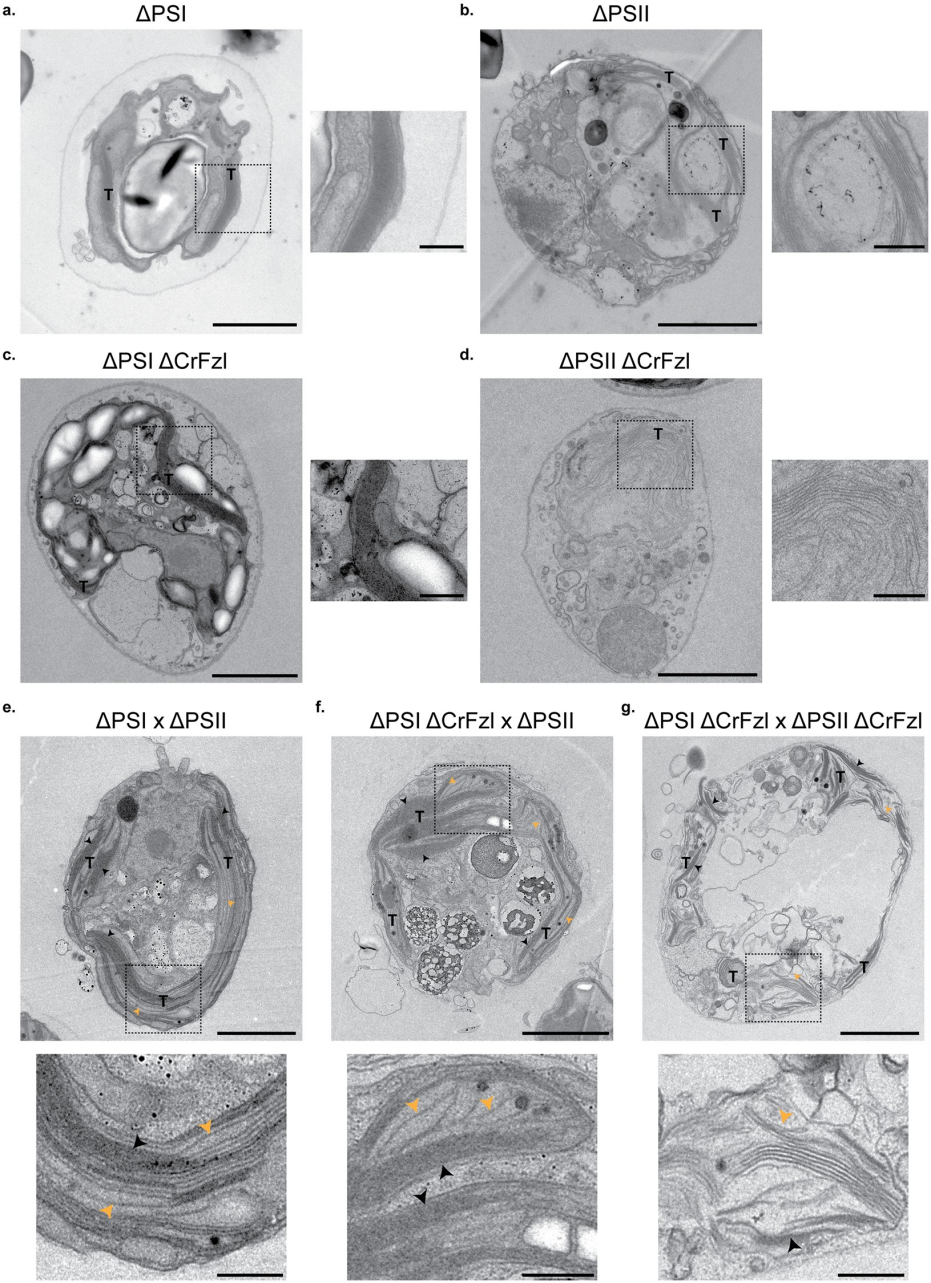
(a-d) Electron micrographs of ΔPSI (a), ΔPSII (b), ΔPSI ΔCrFzl (c) and ΔPSII ΔCrFzl (d) gametic cells. Thylakoids are annotated with a T and the dashed square delimits the enlarged area on the right panel. Scale bar, left panel: 2 μm; right panel: 500 nm. (e-g) Electron micrographs of zygotes from ΔPSI x ΔPSII (e), ΔPSI ΔCrFzl x ΔPSII (f), ΔPSI ΔCrFzl x ΔPSII ΔCrFzl (g) crosses. Thylakoids are annotated with a T and the dashed square delimits the enlarged area in the bottom panel. Black and orange arrowheads point to stacked and loose thylakoids, respectively. Scale bar, top panel: 2 μm; bottom panel: 500 nm. Two more cells of each kind of gamete and zygote are shown in S6 and S7 Figs.

Electron microscopy analysis previously revealed that the thylakoids of ΔPSI and ΔPSII gametes have very discernable characteristics in their stacking properties [39]. In agreement with these observations, we easily noticed that the thylakoids of ΔPSI cells (14 cells; S1 Table) are highly stacked and electron-dense (Fig 7a, S6a and S7a Figs) whereas those from ΔPSII cells (6 cells; S1 Table) are much less stacked with more discernable lumens (Fig 7b, S6b and S7b Figs). Interestingly, the ΔPSI ΔCrFzl and ΔPSII ΔCrFzI gametes kept these differences but also accumulated additional specific features. Together with their highly stacked thylakoids, all the ΔPSI ΔCrFzl gametes that could be analyzed (34 cells; S1 Table) presented a significant increase in starch granules (Fig 7c, S6c and S7c Figs), consistent with the phenotype of vegetative ΔCrFzl mutants (Fig 4a). In contrast, starch granules did not accumulate in the ΔPSII ΔCrFzl gametes (12 cells; S1 Table). However, all cells had thylakoids that were less stacked but also curved (Fig 7d). Remarkably, this curvature often (9 cells out of the 12; S1 Table) resulted in thylakoids organized in concentric stacks similar to fingerprints or onion rings (S6d and S7d Figs). Intriguingly, this phenotype is reminiscent of the fuzzy onion mitochondrial morphology previously observed in early post-meiotic spermatids from mitofusin mutant flies [43].

All the zygotes resulting from ΔPSI and ΔPSII (Fig 7e, S6e and S7e Figs; 23 zygotes observed, S1 Table) or ΔPSI ΔCrFzl and ΔPSII (Fig 7f, S6f and S7f Figs; 4 zygotes observed, S1 Table) crossings in the presence of CAP displayed both highly (black arrowheads) and less stacked thylakoids (orange arrowheads). Nonetheless, the two populations were organized in obvious continuity yielding long and properly assembled thylakoid networks, suggesting efficient thylakoid fusion as expected from fluorescence induction kinetics measurements (Fig 6f). On the other hand, in the absence of CrFzl (10 zygotes observed, S1 Table), the network of intra-plastidial membranes was disorganized (Fig 7g, S6g and S7g Figs). In all zygotes, highly stacked thylakoids (black arrowheads) were mixed with less stacked thylakoids (orange arrowheads) but any continuity between both populations was not discernable. This together with fluorescence measurements (Fig 6f) supports that CrFzl is required for the fusion of thylakoids.

## Discussion

Previous studies performed in the plant model *Arabidopsis thaliana* demonstrated that the absence of Fzl is responsible for a slow growth phenotype associated with pale green or partly chlorotic leaves [20,22], enlarged chloroplasts containing morphologically altered thylakoids [20] as well as activation of autophagy and cell death pathways [22,23]. In addition, photosynthetic impairment was observed in pale green leaves [21]. However, these phenotypes were obtained from distinct mutants and ecotypes which hampered the establishment of any mutual causality between the heterogeneous defects caused by the absence of Fzl expression. Moreover, the primary role of Fzl in maintenance of chloroplast and thylakoid morphology was clearly demonstrated but its precise function in the remodeling of plastidial membranes remained to be addressed. We thus aimed at clarifying the function of Fzl by shifting its analysis in the unicellular microalgal model *Chlamydomonas reinhardtii*.

Our data confirmed the expression and plastidial localization of CrFzl (Fig 1). Using the CRISPR Cas9 technology in *Chlamydomonas reinhardtii*, we generated ΔCrFzl strains with a surprisingly high efficiency (Fig 2) when compared to previous results [31], which might be explained by the absence of cell wall in our recipient strain, our much shorter donor DNA or the nature of our target gene. Phenotypic analysis revealed a strong sensitivity of the mutants to high light stress with decreased resistance to photoinhibition of photosynthesis, which was rescued by antioxidants (Fig 3). An intriguing observation was the increased sensitivity of the CrFzl mutant in mixotrophic (TAP) as compared to photoautotrophic (TP) conditions upon high light stress (Fig 2i and 2j). Interestingly, LHCSR3, one of the major energy dissipating protein in stressful conditions, is far less expressed in mixotrophic than in photoautotrophic conditions upon high light [44]. Whether ΔCrFzl cells have a distinct ability to dissipate excess energy according to their growth in TAP or in TP could thus be tested in the future.

In response to high light treatment, the morphology of ΔCrFzl chloroplasts was also altered with increased starch granules inserted between thylakoids, a marked accumulation of cytosolic vesicles in close physical contact with plastids and an activation of autophagy (Fig 4). This overall sensitivity to light stress allows reconciling the distinct phenotypes previously observed in *Arabidopsis thaliana*. The absence of CrFzl which causes light stress impairment of photosynthesis is likely associated with elevated reactive oxygen species that induce photo-oxidative damage to thylakoid membranes [34]. This extreme damage would then promote the selective degradation of the chloroplast content by cytoplasmic vacuoles and ultimately cell death, upon prolonged exposure to high light. Most importantly, the defective resistance to photo-inhibition that induces this cascade of events may result from abrogation of CrFzl primary function in mediating fusion of thylakoids.

We should at this point not exclude that CrFzl could modulate membrane organization and stabilization but our work indicates that Fzl may trigger the fusion of thylakoid membranes. In conditions where thylakoid fusion is required, zygotes from a cross between ΔPSI and ΔPSII strains lacking CrFzl failed to recover photosynthetic activity (Fig 6) and presented disorganized thylakoid networks (Fig 7). Conversely, when CrFzl was expressed in only one of the two strains to be mated, ΔPSI and ΔPSII thylakoids remained competent for mixing their membrane content as attested by their recovered photosynthetic activity (Fig 6) and the proper morphology of their thylakoid networks (Fig 7). The most straightforward interpretation for this thylakoid fusion occurring after mating of CrFzl positive and negative strains relies on our data suggesting that CrFzl is soluble in the stroma of the chloroplast (Fig 1). In this context, the CrFzl protein expressed in one of the two chloroplasts could diffuse and bind thylakoids of the ΔCrFzl strain. The DRP present on both sets of thylakoids would then promote their tethering and subsequent fusion. Yet, fusion DRPs such as mitofusins or OPA1 are integral to mitochondrial outer and inner membranes, which contrasts with CrFzl being essentially soluble in the stroma of the chloroplast. In fact, such feature is actually shared by NpBDLP, for which insertion into membranes is triggered by a GTPase domain-dependent conformational switch [12,13]. NpBDLP includes a membrane paddle instead of a regular transmembrane domain. In the apo or GDP bound form of BDLP, this paddle is not competent for membrane binding but becomes accessible upon the binding of GTP that induces the conformational switch of the DRP. Consistent with its essential GTPase and HD domains, CrFzl may function in a similar fashion and our data further suggest that the targeting to thylakoid membranes is stimulated upon light stress (Fig 5). We found that the GTPase and HD region both regulate this light-dependent recruitment of CrFzl to thylakoids (Fig 5g). Deletion of the HD inhibited the association of CrFzl to membranes under high illumination. While this likely reflects the requirement of the HD for CrFzl recruitment to membranes, we cannot exclude that this effect was caused by a disturbed structure of the mutant. On the other hand, the G1 mutation surprisingly stimulated the binding of CrFzl to thylakoids upon high light. Whether this G1 mutation (K448M) abolishes the binding of GTP or abrogates GTP hydrolysis is currently unclear. However, similar to BDLP [12,13], binding of GTP in the absence of hydrolysis may maintain CrFzl in a conformation competent for stable membrane association. Moreover, since the G1 mutant only responded to high but not low illumination (Fig 5g), an additional light stress-dependent signal may participate in the recruitment of CrFzl to thylakoids. In this regard, CrFzl has recently been identified as a thioredoxin target [45], suggesting that CrFzl-mediated membrane fusion induced by high light treatment may be redox regulated by the opposing actions of ROS and thioredoxin, as previously established for the regulation of autophagy [46,47].

Intriguingly, obvious phenotypes in thylakoids from Crfzl mutants were not observed in vegetative conditions. In this regard, Fzl-mediated fusion may occur in between thylakoid stacks [24]. This may explain that defects in thylakoid organization caused by the absence of CrFzl could only be observed in cells with weak thylakoid stacking (compare thylakoids from Figs 4d and 7c Vs those from Fig 7d and S6d Fig). The mating assay we employed indicates that zygotes lacking CrFzl do not fuse their thylakoids. In this context, fusion of thylakoids is not essential for growth in “normal” conditions but becomes required during light stress. This echoes the stress-related function attributed to the NpBDLP paralogue of *Bacillus subtilis*, DynA, which has been implicated in the repair of membrane pores formed by antibiotics [48]. In the case of CrFzl, promoting fusion between two distinct thylakoid saccules during light stress could participate in driving a continuous redistribution of functional photosystems between thylakoids, thus maintaining homogenous photosynthetic electron transfer. In this scenario, CrFzl would avoid the accumulation of thylakoids with very low photosynthetic efficiency that may otherwise produce significant amounts of ROS. The absence of thylakoid fusion may thus lead to such damages that the cells would induce the selective degradation of chloroplast material to cope with further insults resulting from photosynthetic dysfunction. The cytoplasmic vesicles accolated to chloroplasts (Fig 4, S3 Fig) and the activation of autophagy (Fig 4g) that we observed in this study give credence to this possibility. However, the mechanism of formation of these vesicles and their relationship to autophagy remains obscure, which warrants future investigations.

While CrFzl triggers thylakoid fusion during light stress, outer and inner envelops of chloroplasts must also fuse during mating. EZY8 (Early Zygote 8; Cre06.g250650) represents a good candidate for this function as microarray and RNA-Seq studies detected increased expression of this DRP during early stages of zygote development [49,50]. In parallel, Fzl proteins are the only mitofusin-like proteins in plants and this study further confirms that they do not participate in mitochondrial dynamics. Yet, several studies have demonstrated that mitochondrial fusion takes place in plant cells and microalgae [51,52,53]. Future discoveries will likely resolve this fascinating issue and extend the contribution of DRPs to the control of chloroplast membrane dynamics.

## Materials and methods

### Strains and media

The strain used for initial expression of CrFzl and fluorescence subcellular localization was CC-4533 [27]. The strain used for subcellular fractionation and mutagenesis and as a wild-type control in all following experiments was cw15.J3 (*mt-*). As a control for CrFzl knock-out in the screening of CRISPR Cas9 generated strains, the mutant LMJ.RY0402.175738, containing a paromomycin cassette insertion in the *CrFzl* locus, was obtained from the CLiP library [27]. Cells were maintained on TAP agar 1,5% containing the appropriate antibiotic if necessary, and inoculated in TAP for preculture prior to any experiment. Unless otherwise stated, cultures were grown in continuous light at 50 μmol photons.m^−2^·s^−1^, 26°C on a rotary shaker at 120 rpm. For minimal medium cultures, the TP medium is the same composition as TAP but with pH adjusted with HCl instead of acetate.

Crosses required to obtain ΔPSI ΔCrFzl *mt+*, ΔPSII *mt-* and ΔPSII ΔCrFzl *mt-* strains were performed according to [25] and are described in the “Thylakoid fusion assay” section of materials and methods.

### Bioinformatic of CrFzl and phylogenetic analysis of Dynamin Related Proteins

Chloroplast transit peptide length was predicted using Wold PSort [54]. CrFzl sequence was blasted in SMART [55] for subdomain prediction and Phobius [56], TMHMM2.0 [57], CCTOP [58], PredictProtein [59] and TCDB [60], for transmembrane subdomain prediction. Three out of 5 online programs predicted two transmembrane domains (CCTOP: 921–940 and 948–965; TCDB: 922–938 and 948–968; PredictProtein: 924–943 and 947–959) while Phobius only predicted one (948–967), and TMHMM2.0 none. Protein sequences for different DRPs have been collected from Phytozome (for Arabidopsis and Chlamydomonas proteins), SGD (for Yeast) and UniProt (identifiers: Ns_BDLP, B2IZD3; Hs_Mfn1, Q8IWA4; Hs_dynamin, Q05193; Hs_dynamin-like, O00429; Hs_MxA, P20591; Hs_OPA1, O60313; Hs_GBP1, P32455). GTPases domains were delimitated using SMART prediction and all remaining sequences were analyzed on ClustalW online Software.

### Plasmid construction

Cloning of *Crfzl* locus into pSL26 was done using NEBuilder HiFi DNA Assembly Cloning Kit with 4 fragments. Backbone vector was produced by restriction of pSL26 with NdeI/NruI. 5’ and 3’ “adaptor” fragments were synthesized by PCR using the primer pairs CrFzl_ATG_For/CrFzl_ATG_Rev and CrFzl_STOP-HA_For/CrFzl_STOP_HA_Rev, respectively, and the base pairs from +711 to +6199 of the *CrFzl* locus were excised from BAC vector PTQ13345 using a SphI/BciVI restriction reaction. The same strategy was adopted for cloning into pLM005-CrVenus vector, using different 5’ forward and 3’ reverse primers containing sequence homologous to the backbone vector (See S2 Table).

For construction of the mutated CrFzl versions, the plasmid for expression of the full length CrFzl-3HA was digested with AgeI (insertion of the GTPase domain mutation), or MluI/NruI (deletion of the TM and CC domains). The CrFzl region containing the G1-box of the GTPase domain was amplified with the nucleotide substitution A to T at position +2709 (AAG→ATG) using the primers pairs PreG1_For/Pre-G1_Rev and Post-G1_For/Post-G1_Rev. 3’ part of the gene without the TM regions was amplified with Pre-TM_For/Pre-TM_Rev and Post-TM_For/Post-TM_Rev. The ΔCC fragment was produced using the pair Pre-TM_For/ΔCC_Rev.

### Chlamydomonas transformation

Cells in mid-log phase were centrifuged and resuspended in TAP medium containing 40 mM sucrose at a density of ~2.10^8^ cells.mL^-1^. 250 μL aliquots were transferred to 4 mm electroporation cuvettes and incubated for 20 mn on ice. 500 ng of linearized plasmid were added and the suspension was homogenized by flicking the cuvette. A time constant electric pulse of 800V was then applied (11 ms, 800 V, Xcell Pulser Electroporation System, Biorad). Cells were left for 5 mn at room temperature, diluted in TAP 40 mM sucrose and incubated for a 18 hours recovery in low light with agitation. After recovery, the cells were pelleted, resuspended in fresh TAP sucrose and plated on TAP medium containing paromomycin (10 μg.mL^-1^). Resistant clones were allowed to grow for 6 days before picking, ordering and screening.

### Western blot

One OD_750nm_ (1 mL) of cells was lyzed with 100 μL of 1 M NaOH for 10 mn on ice then proteins were precipitated by addition of 100 μL of 50% TCA on ice for 30 mn. Proteins were then pelleted, resuspended in Sample Buffer (1.6 mM EDTA, 1.6% SDS, 40 mM DTT, 8% Glycerol, 0.016% Bromophenol Blue, 333 mM TrisBase) and solubilized at 70°C for 10 mn prior to loading. After SDS-PAGE, proteins were transferred onto a nitrocellulose membrane and detected with specific commercial (anti-RbcL, anti-NAB1 and anti-βCF1 from Agrisera, anti-HA [clone 12ca5] and anti-Tub from Sigma-Aldrich) and custom made (anti-CrFzl from Covalab) primary antibodies. Unless otherwise stated, 10 μL of protein extract were loaded and Tubulin antibodies served as loading control.

### Subcellular fractionation

Chloroplast isolation was performed as described in [26] with some modifications. Briefly, mid-log phase cells (OD_750nm_ ~ 0,8) were concentrated to 0.7 mg chlorophyll per mL in isolation buffer (HEPES 50 mM, Sorbitol 300 mM, EDTA 2 mM, MgCl_2_ 1 mM, pH 7,5). Cells were then broken by passing through a 27 gauge needle with a flow of ~0.1 mL.s^-1^. Unbroken cells and chloroplasts were pelleted by centrifugation at 800 g, 4°C for 5 mn. Supernatant was collected as the cytosolic fraction. The pellet was gently resuspended in isolation buffer using a fine paintbrush. The chloroplast suspension was then layered on top of a 3 cautions Percoll gradient (20%/45%/65%) and centrifuged at 5525 g, 4°C for 30 mn with slow acceleration and no brake. While unbroken cells cross the three cautions and pellet at the bottom of the gradient, the thylakoid enriched fraction was recovered at the 20/45% Percoll interface and intact chloroplasts were harvested from the 45/65% interface. Both fractions were washed from Percoll in 50 mL isolation buffer and centrifuged at 700 g, 4°C for 3 mn. Pelleted chloroplasts were collected and frozen while the thylakoid fraction was split in four sub-fractions. Those sub-fractions were further washed and pelleted twice in 5 mL HEPES 50 mM, Sorbitol 300 mM containing 0, 50, 150 or 500 mM NaCl, respectively; and centrifuged at 2759 g, 4°C for 10 mn. Thylakoid pellets were finally frozen. Proteins were then extracted as described above (Western blot) and loading was adapted to obtain equivalent amounts of thylakoid protein marker (β-CF1) in the total, chloroplast and thylakoid fractions.

For membrane/soluble fractions separation, cells were broken in HEPES 50mM by ten cycles of 10 s shaking/15 s on ice with glass beads (diameter 425–600 μm) in a cold room. Unbroken cells were pelleted by centrifugation at 800 g, 4°C for 5 mn. The supernatant was then further centrifuged at 21000 g, 4°C for 30 mn. Resulting supernatant containing soluble proteins and pellet containing membrane proteins were harvested and proteins extracted as described above.

### Fluorescence microscopy

After treatment, cells were fixed by adding formaldehyde at a final concentration of 3,7% and incubated at room temperature with agitation for 30 mn. Cells were mounted between glass slide and coverslips after 2 subsequent washes with TAP medium. Imaging was performed with a Zeiss Axio Observer.Z1 microscope (Carl Zeiss S.A.S.) with a X100 oil immersion objective equipped with the filter sets 10 Alexa Fluor (Excitation BP 450/490, Beam Splitter FT 510, Emission BP 515–565), DAPI (Excitation BP 359–371, Beam Splitter FT 395, Emission BP 397-∞), 47 HE CFP (Excitation BP 424/448, Beam Splitter FT 455, Emission BP 460–500), 46 HE YFP (Excitation BP 488–512, Beam Splitter FT 515, Emission BP 520–550) and Chloro (Excitation BP 450/490, Beam Splitter FT 505, Emission BP 600-∞). Cell contours were visualized with Nomarski optics. Images were acquired with an ORCA-R2 charge-coupled device camera (Hamamatsu) and analyzed with ImageJ.

### Antibody production

Custom made antibodies were ordered from Covalab. Anti-CrFzl antibodies were produced by immunization of a rabbit with two synthesized peptides (peptide 1 [289–304]: C-FDLAENATAEDYAQA-coNH2 and peptide 2 (727–741): C-GRQLGRFRAEMEKDA-coNH2).

### CRISPR-Cas9 mediated mutagenesis

Protocol for targeted disruption of Cre14.g616600 locus was adapted from Shin *et al*., 2016 [31]. Cells were transformed as described above. Prior to the electric pulse, sgRNA (33,33 μg.mL^-1^) and purified Cas9 protein (25 μg.mL^-1^, Labomics), preincubated at 37°C for 30 min, and a DNA cassette (1 μg.mL^-1^) containing the *aph7”* gene conferring resistance to hygromycin, were added to the 250 μL aliquots. After recovery, the cells were pelleted, resuspended in fresh TAP sucrose and plated on TAP medium containing hygromycin (20 μg.mL^-1^).

The hygromycin cassette was amplified from the pSLHyg plasmid using the primers HygFor and HygRev, and the Phusion High-Fidelity polymerase (NEB) following the manufacturer’s instructions with an annealing temperature of 70°C and 40 s of extension for 35 cycles.

Hygromycin resistant clones were screened for targeted cassette insertion using a 3 primers PCR strategy. After quick DNA extraction by boiling in 50 μL of 10 mM EDTA, 1 μL of supernatant was used for PCR reaction with primers CrFzl_ATG_For, CrFzl_ATG_Rev and Hyg_500_Rev with hybridization temperature set at 60°C and polymerization allowed for 90 s. PCR conditions are set such as three possible products are expected depending on the insertion of the cassette in the GOI and its orientation: 760 bp from the pair CrFzl_ATG_For/CrFzl_ATG_Rev, 1246 bp from CrFzl_ATG_For/Hyg_500_Rev and 715 bp from CrFzl_ATG_Rev/Hyg_500_Rev.

### Growth assay

For spot tests, Chlamydomonas cells in log-phase where harvested by centrifugation at 1500g for 5 mn. They were then washed one time with minimal medium before being resuspended at 2 OD_750nm_/mL. Drops (3 μL) were deposited on minimal TP and enriched TAP media and grown at 50, 100 and 200 μmol photons.m^−2^·s^−1^ for 5 days.

For liquid cultures, mid-log phase cells were inoculated in fresh TP or TAP medium at a starting OD_750nm_ of 0.1. Growth in darkness and at 50 and 400 μmol photons.m^−2^·s^−1^ was monitored by measuring optical density at 750 nm.

### Rhodamine 123 staining

Chlamydomonas mid-log cells were harvested and Rhodamine 123 added at a final concentration of 25 μM. Cells were incubated for 25 mn at RT, washed twice and concentrated in TAP medium before observations.

### Light stress

For photoinhibition measurements, mid-log phase cells from wild-type and mutant background were diluted in TAP or TP medium at OD_750nm_ = 0.15, left for 2 hours to adapt to the fresh medium and exposed to high light (850 μmol photons.m^−2^·s^−1^) for 1 hour. They were then left for recovery in dim light (< 5 μmol photons.m^−2^·s^−1^) for 3 h. PSII efficiency (F_v_/F_m_) was measured every 30 mn for the first two hours and every hour thereafter.

For detection of fluorescent dots and fluorescence microscopy study of CrFzl membrane association, cells inoculated at an OD_750nm_ = 0.15 were incubated for 6 hours at 400 μmol photons.m^−2^·s^−1^. Treated cells were immediately fixed following light stress as described previously.

For the biochemical study of CrFzl membrane association, cells were grown to OD_750nm_ = 0.4 and treated for 6 hours at 800 μmol photons.m^−2^·s^−1^ before proceeding to cellular subfractionation.

### Fluorescence measurements

Fluorescence measurements were performed using a DeepGreen Fluorometer (JBeamBio). PSII efficiency, Fv/Fm (Fv = Fm − F0), was determined using shortly (1 mn) dark-adapted cells. F0 is the initial chlorophyll fluorescence measured after dark adaptation and Fm is the maximum fluorescence measured after a brief and saturating flash of green light [61,62].

### Antioxidant treatment

Cells of wild-type and mutant background were inoculated at OD_750nm_ = 1 in TAP medium containing 0, 1, 2.5 or 5 mM N-acetyl cysteine (NAC; buffered with 100 mM HEPES, pH 7.5); and treated for 24 hours with high light stress at 800 μmol photons.m^−2^·s^−1^. As a control, the NAC-free cultures were duplicated and grown at normal light conditions (50 μmol photons.m^−2^·s^−1^).

### Electron microscopy

Cells incubated in darkness, normal or high light for 6 hours, as well as gametic cells and zygotes from the crosses were concentrated, deposited on polylysine-coated cover slips, fixed with a mixture of 2% (wt/vol) paraformaldehyde, 1% (wt/vol) glutaraldehyde in 0.2 M phosphate buffer (PB), pH 7.4, post-fixed with 1% (wt/vol) OsO4 supplemented with 1.5% (wt/vol) potassium ferrocyanide, dehydrated in ethanol, and embedded in epon 812 (TAAB Laboratories Equipment). Ultrathin sections were prepared with a Reichert UltracutS ultramicrotome (Leica), counter-stained with uranyl acetate, and viewed at 80kV with a Transmission Electron Microscope (TEM; Tecnai Spirit G2; Thermo Fisher Scientific, Eindhoven, The Netherlands) equipped with a QUEMESA CCD 4K camera (EMSIS GmbH, Münster, Germany) using iTEM software (EMSIS).

### ATG8 induction

As a control of ATG8 induction, the wild-type cw15 strain (OD_750nm_ = 0.25) was treated with 0.5 and 1 μM rapamycin for 16 hours. In parallel, wild-type and ΔCrFzl strains were treated for 6 hours at 400 μmol photons.m^−2^·s^−1^ at OD_750nm_ = 0.15. Proteins were then extracted as described in [63]. 20 μg of proteins were loaded for each sample.

### Thylakoid fusion assay

Strains required for this assay were obtained by cross the ΔPSI mutant F15 (*mt+*) and the ΔPSII mutant Fud34 (*mt+*) with the ΔCrFzl strain (*mt-*). Zygotes were germinated in TAP sorbitol containing 2% sorbitol in dim light for 24 hours. Aliquot were then spread on agar plates containing Hygromycin B (20 μg.mL^-1^) to select for strain defective for CrFzl. Single colonies were then picked and arrayed on new agar plates before measurements of chlorophyll induction kinetics. Clones displaying a fluorescence kinetic typical of ΔPSI or ΔPSII mutants were selected for mating type identification by PCR [64].

For the thylakoid fusion assay, induction of gametogenesis was done by an overnight incubation in nitrogen deprived medium under dim light. For each cross, parental gametes were mixed in equivalent amount of cells to reach a total of ~1,5.10^7^ cells.mL^-1^. Chloramphenicol was added simultaneously at a concentration of 100 μg.mL^-1^. For antibiotic-free control crosses, ethanol, in which CAP is solubilized, was added to a final concentration of 0,3%. Crosses were left to process at 50 μmol photons.m^−2^·s^−1^ without agitation for 48 hours which represents the light phase of zygote development. The zygote pellicle deposited at the bottom of Petri dishes was washed from gametic cells and the zygotes resuspended in water containing 2% sorbitol with or without CAP prior to fluorescence measurements, which were performed using a DeepGreen Fluorometer after dark adaptation of the cells (1 mn).

## Supporting information

S1 Fig (a) Subcellular fractionation of chloroplasts and thylakoids from a CrFzl-3HA expressing strain. Thylakoid β-F1 subunit of the ATPase complex (β-CF1), the large subunit of the RuBisCo (RbcL) and Nucleic Acid Binding protein (NAB1) were used as marker antibodies for thylakoids, chloroplast and cytosol proteins, respectively. Loadings were adapted to reach similar amounts of β-CF1. (b) Subcellular fractionation of chloroplasts and thylakoids from a wild-type strain. Thylakoid β-CF1 subunit of the ATPase complex (β-CF1), the large subunit of the RuBisCo (RbcL) and Nucleic Acid Binding protein (NAB1) were used as marker antibodies for thylakoids, chloroplast and cytosol proteins, respectively. Loadings were adapted to reach similar amounts of β-CF1. This is a longer exposure for the CrFzl immunoblotting displayed in Fig 1f which shows the appearance of a band higher than CrFzl in total and cytosolic extracts (asterisk).
(PDF)

S2 Fig Overview of the targeted mutagenesis strategy. (a) The intron/exon organization of the Cre14.g616600 locus is represented. Hybridization of the single guide RNA with its specific target sequence induces a cleavage by the Cas9 nuclease between nucleotide 66 and 67 of the first exon. (b) Schematic representation of the expected genetic organization of the *CrFzl* locus before and after insertion of the *aph7”* cassette. CrFzl primers CrFzl-ATG-F and CrFzl-ATG-R allow the amplification of the wild-type 760 bp fragment. After transformation and insertion of the hygromycin cassette at the site of targeted mutagenesis, the use of the third primer Hyg500-R lead to the amplification of a 715 bp product in the case of a sense insertion, or 1261 bp in the case of an antisense insertion. (c) Sequences of hygromycin cassette insertion in Cas9 target site for 4 CrFzl knock-out strains (#1, #8, #10, #12). Nucleotides surrounding the initial cut site are represented in black while 5’ and 3’ extremities of the hygromycin cassette are displayed in red. Note that, in clone #1, the shorter size of the PCR product seen in Fig 2c is due to a small deletion of 187 nucleotides at the end of the cassette but after the terminator sequence. In clone #12, a small sequence GTAGTG (blue) from unknown origin was inserted between the genomic DNA and the resistance cassette during the insertion.
(PDF)

S3 Fig (a) *Top panel*. Fluorescence microscopy of wild-type and ΔCrFzl #1 chloroplasts after 6 hours of normal light (NL). Chlorophyll autofluorescence (Chl) is used to analyze chloroplast morphology. Scale bar: 2 μm. *Bottom panel*. Large fluorescence microscopy field of wild-type and ΔCrFzl #1 chloroplasts after 6 hours of normal light (NL) or high light (HL). Chlorophyll autofluorescence (Chl) is used to analyze chloroplast morphology. Scale bar: 5 μm. (b) *Top 6 panels*. Fluorescence microscopy of ΔCrFzl #1 cells after 6 hours of light stress. Images were taken with wavelength/filter sets used for detection of DAPI, YFP, CFP and chlorophyll (Chl). Scale bar: 5 μm. Arrowheads indicate auto-fluorescent dots detected with the DAPI, YFP and CFP filter sets. *Bottom 4 panels*. Fluorescence microscopy of ΔCrFzl #1 cells after 6 hours of light stress. Images were taken with wavelength/filter sets used for detection of GFP and chlorophyll (Chl). Scale bar: 5 μm. Arrowheads indicate auto-fluorescent dots detected with the GFP filter set. (c) Electron micrographs of wild-type and ΔCrFzl #1 cells exposed to normal light (left panel) and high light (right panel). cp: chloroplast; p: pyrenoid; arrows/v: vesicles; asterisks: extra-pyrenoidal starch granules. Scale bar: 2 μm. (d) Percentage of cells displaying a normal pyrenoid structure in wild-type and ΔCrFzl #1 cells during darkness, normal and high light conditions. Error bars represent the s.e.m. from the number of analyzed cells in WT Darkness: 71; WT NL: 91; WT HL: 56; ΔCrFzl Darkness: 93; ΔCrFzl NL: 88; ΔCrFzl HL: 69. ** p < 0,05. *** p < 0,005 (Student test).
(PDF)

S4 Fig (a) Fluorescence microscopy of cells from the wild-type (top) and a ΔCrFzl strain expressing the CrFzl-mVenus construct (bottom) after growth in normal (NL, left) and high light (HL, right) conditions. Chlorophyll autofluorescence is used as a chloroplast marker. Scale bar: 5 μm. (b) Percentage of cells with fluorescent dots in normal (NL) and high light (HL) conditions in the wild-type (blue), the ΔCrFzl (red) and the ΔCrFzl strains expressing CrFzl-3HA (top left, orange), CrFzl-G1*-3HA (top right, dark green), CrFzl-ΔHD-3HA (bottom left, green) and CrFzl-ΔCC-3HA (bottom right, light green). Error bars represent the s.d. from three independent experiments. ** p < 0,05. *** p < 0,005 (Student test). (c) Western blot analysis of CrFzl-3HA, CrFzl-G1*-3HA and CrFzl-ΔHD-3HA in thylakoid fractions washed with 0, 50, 150 and 500 mM NaCl after 6 hours of normal (NL) or high light (HL) treatment. Loadings were adapted to reach similar amounts of β-CF1. Variations in CrFzl-HA in thylakoid washed with 50 and 150 mM NaCl are much less significant than at 500 mM and were then not shown in Fig 5f.
(PDF)

S5 Fig Chlorophyll fluorescence induction kinetics from zygotes of the ΔPSI ΔCrFzl x ΔPSII ΔCrFzl cross performed in the absence or in the presence of CAP. Two additional crosses (mating 2 and 3) are represented.
(PDF)

S6 Fig (a-d) Electron micrographs of ΔPSI (a), ΔPSII (b), ΔPSI ΔCrFzl (c) and ΔPSII ΔCrFzl (d) gametic cells. Thylakoids are annotated with a T and the dashed square delimits the enlarged area on the right panel. Scale bar, left panel: 2 μm; right panel: 500 nm. (e-g) Electron micrographs of zygotes from ΔPSI x ΔPSII (e), ΔPSI ΔCrFzl x ΔPSII (f), ΔPSI ΔCrFzl x ΔPSII ΔCrFzl (g) crosses. Thylakoids are annotated with a T and the dashed square delimits the enlarged area in the bottom panel. Black and orange arrowheads point to stacked and loose thylakoids, respectively. Scale bar, top panel: 2 μm; bottom panel: 500 nm. Two more cells of each kind of gamete and zygote are shown in Fig 7 and S7 Fig.
(PDF)

S7 Fig (a-d) Electron micrographs of ΔPSI (a), ΔPSII (b), ΔPSI ΔCrFzl (c) and ΔPSII ΔCrFzl (d) gametic cells. Thylakoids are annotated with a T and the dashed square delimits the enlarged area on the right panel. Scale bar, left panel: 2 μm; right panel: 500 nm. (e-g) Electron micrographs of zygotes from ΔPSI x ΔPSII (e), ΔPSI ΔCrFzl x ΔPSII (f), ΔPSI ΔCrFzl x ΔPSII ΔCrFzl (g) crosses. Thylakoids are annotated with a T and the dashed square delimits the enlarged area in the bottom panel. Black and orange arrowheads point to stacked and loose thylakoids, respectively. Scale bar, top panel: 2 μm; bottom panel: 500 nm. Two more cells of each kind of gamete and zygote are shown in Fig 7 and S6 Fig.
(PDF)

S1 Table Cells and associated phenotypes analyzed in the thylakoid fusion mating assay. The total number of gametes (ΔPSI; ΔPSII; ΔPSI ΔCrfzl; ΔPSII ΔCrfzl) and zygotes (ΔPSI X ΔPSII; ΔPSI ΔCrfzl X ΔPSII; ΔPSI ΔCrfzl X ΔPSII ΔCrfzl) analyzed by electron microscopy is indicated. All gametes with the PSI mutation (ΔPSI and ΔPSI Δfzl) showed highly stacked thylakoids whereas those with the PSII mutation (ΔPSII and ΔPSII Δfzl) had less stacked thylakoids. In addition to being less stacked, the thylakoids from PSII Δfzl cells were systematically curved and often organized in fingerprint-like structures. All zygotes expressing Fzl (ΔPSI X ΔPSII and ΔPSI Δfzl X ΔPSII) had highly and less stacked thylakoids in continuity and organized as a proper network. In contrast, zygotes that do not express Fzl (ΔPSI Δfzl X ΔPSII Δfzl) had disorganized thylakoid networks with highly and less stacked thylakoids there were not in continuity.
(PDF)

S2 Table Primers used in this study. (PDF)

S3 Table Strains used in this study. (PDF)
