# Supplementary figures and images for: The dynamin-like protein Fzl promotes thylakoid fusion and resistance to light stress in Chlamydomonas reinhardtii
Source: PLoS Genet. 2019 Mar 15;15(3):e1008047. doi: 10.1371/journal.pgen.1008047 (PMC6436760; doi:10.1371/journal.pgen.1008047)

**a.**

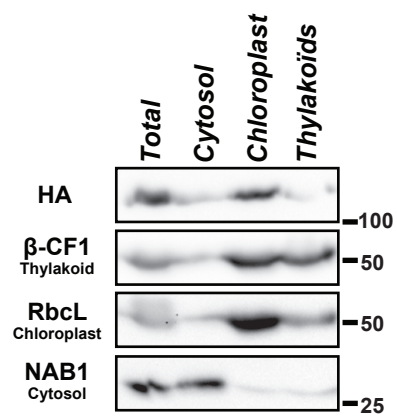

**b.**

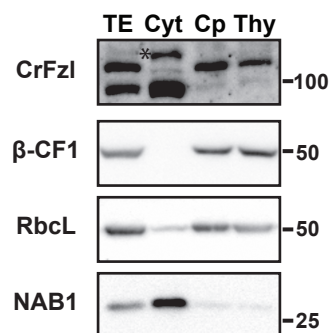

Supplement: S1 Fig — (a) Subcellular fractionation of chloroplasts and thylakoids from a CrFzl-3HA expressing strain. Thylakoid β-F1 subunit of the ATPase complex (β-CF1), the large subunit of the RuBisCo (RbcL) and Nucleic Acid Binding protein (NAB1) were used as marker antibodies for thylakoids, chloroplast and cytosol proteins, respectively. Loadings were adapted to reach similar amounts of β-CF1. (b) Subcellular fractionation of chloroplasts and thylakoids from a wild-type strain. Thylakoid β-CF1 subunit of the ATPase complex (β-CF1), the large subunit of the RuBisCo (RbcL) and Nucleic Acid Binding protein (NAB1) were used as marker antibodies for thylakoids, chloroplast and cytosol proteins, respectively. Loadings were adapted to reach similar amounts of β-CF1. This is a longer exposure for the CrFzl immunoblotting displayed in Fig 1f which shows the appearance of a band higher than CrFzl in total and cytosolic extracts (asterisk). (PDF) [file pgen.1008047.s001.pdf]

**a. *CrFzl* gene organization**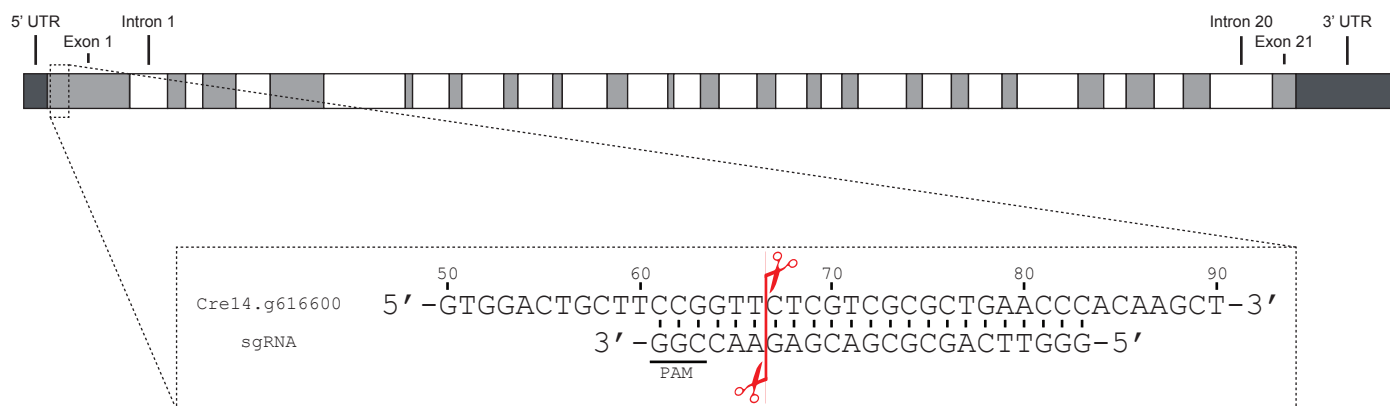**b.**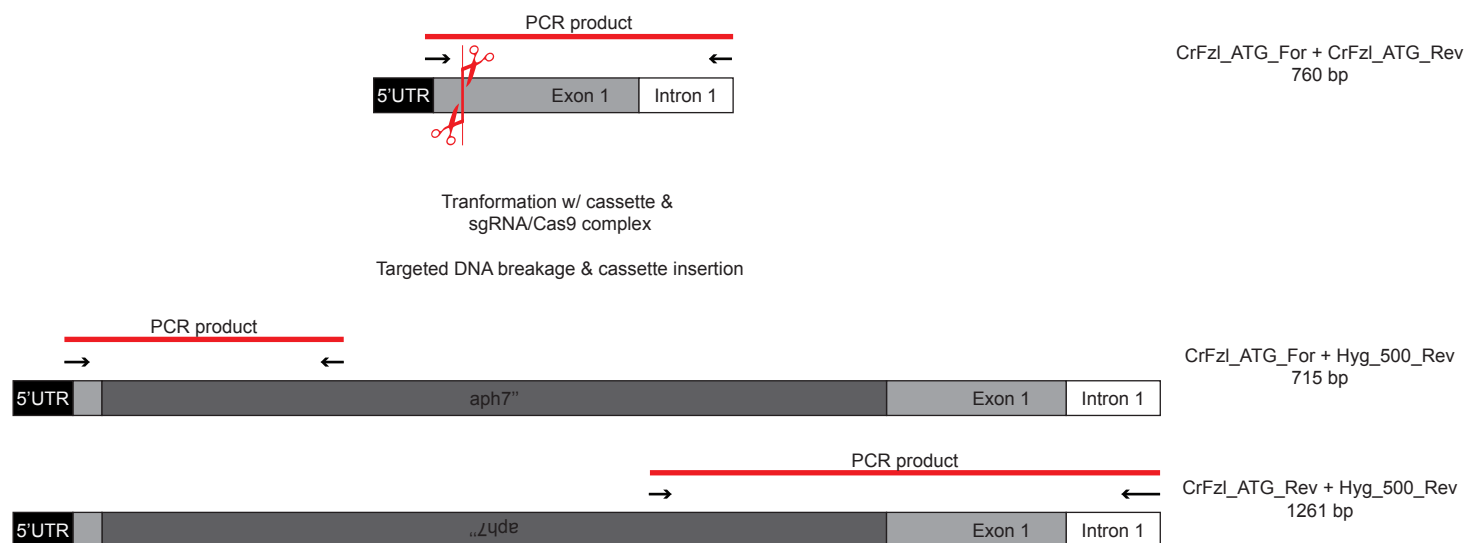**c.**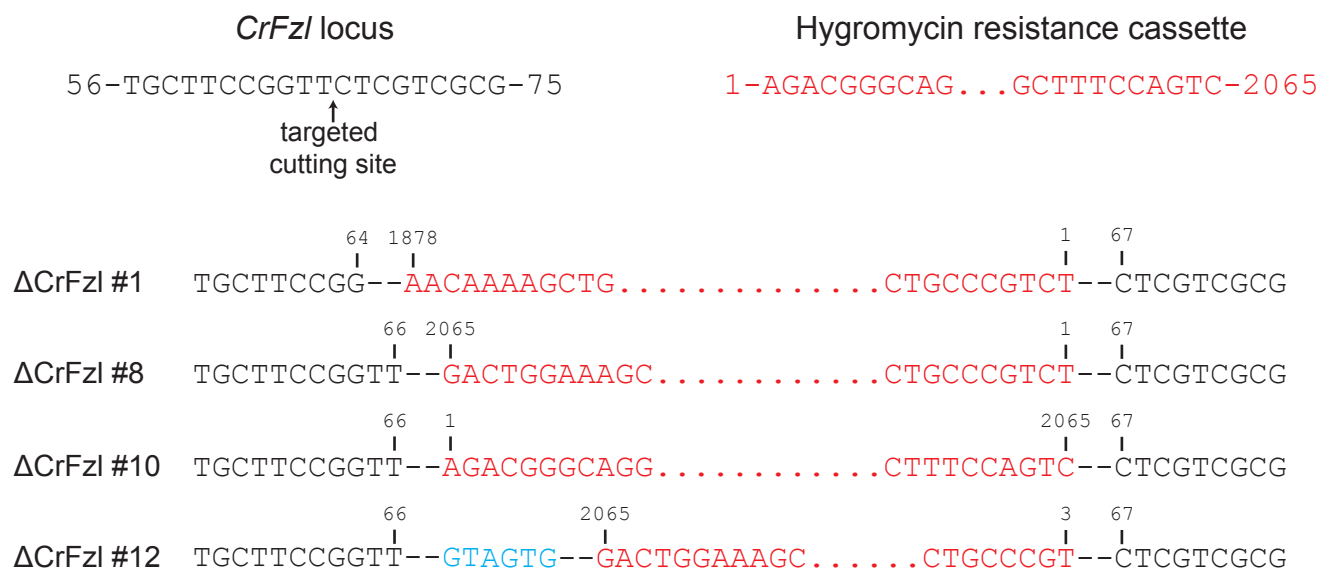

Supplement: S2 Fig — (a) The intron/exon organization of the Cre14.g616600 locus is represented. Hybridization of the single guide RNA with its specific target sequence induces a cleavage by the Cas9 nuclease between nucleotide 66 and 67 of the first exon. (b) Schematic representation of the expected genetic organization of the CrFzl locus before and after insertion of the aph7” cassette. CrFzl primers CrFzl-ATG-F and CrFzl-ATG-R allow the amplification of the wild-type 760 bp fragment. After transformation and insertion of the hygromycin cassette at the site of targeted mutagenesis, the use of the third primer Hyg500-R lead to the amplification of a 715 bp product in the case of a sense insertion, or 1261 bp in the case of an antisense insertion. (c) Sequences of hygromycin cassette insertion in Cas9 target site for 4 CrFzl knock-out strains (#1, #8, #10, #12). Nucleotides surrounding the initial cut site are represented in black while 5’ and 3’ extremities of the hygromycin cassette are displayed in red. Note that, in clone #1, the shorter size of the PCR product seen in Fig 2c is due to a small deletion of 187 nucleotides at the end of the cassette but after the terminator sequence. In clone #12, a small sequence GTAGTG (blue) from unknown origin was inserted between the genomic DNA and the resistance cassette during the insertion. (PDF) [file pgen.1008047.s002.pdf]

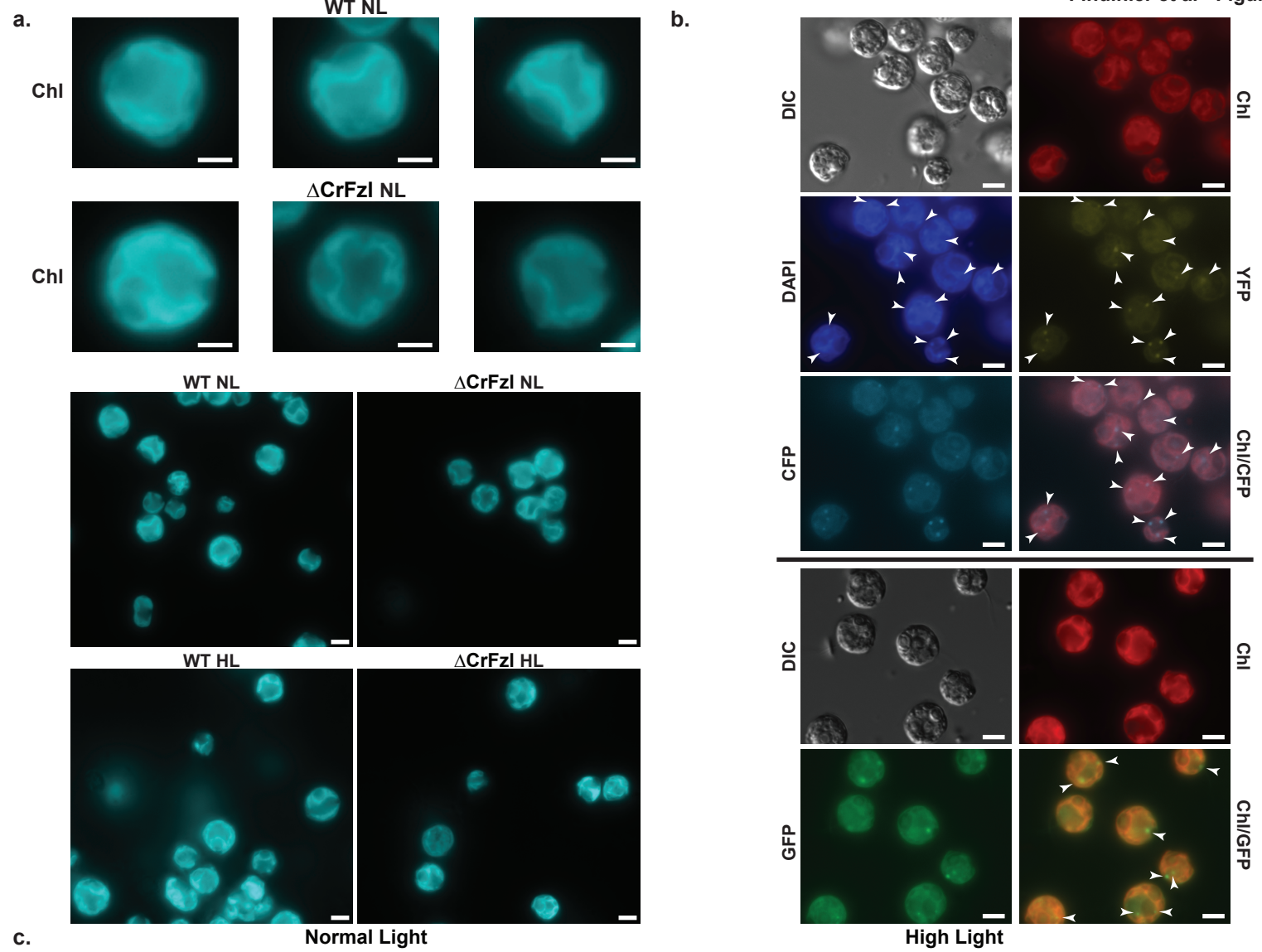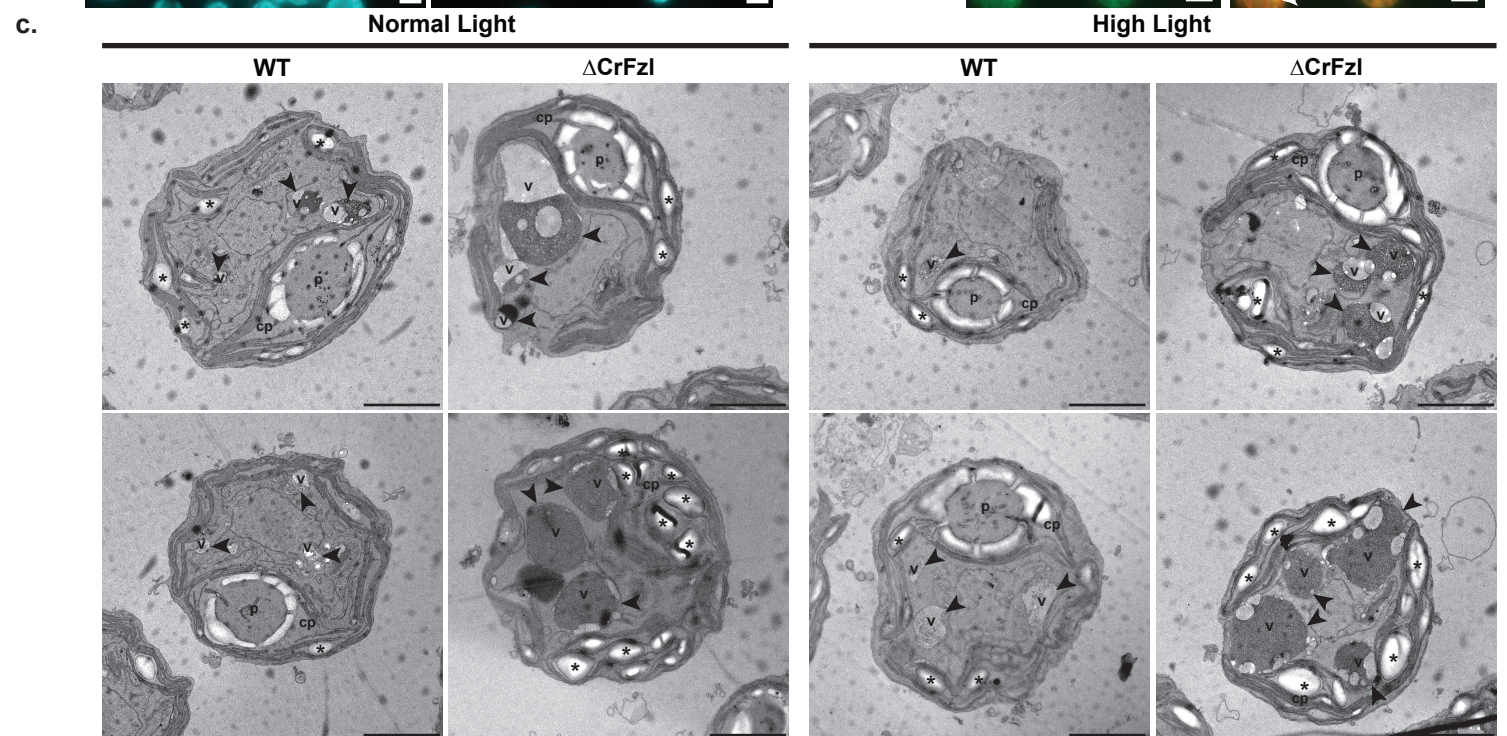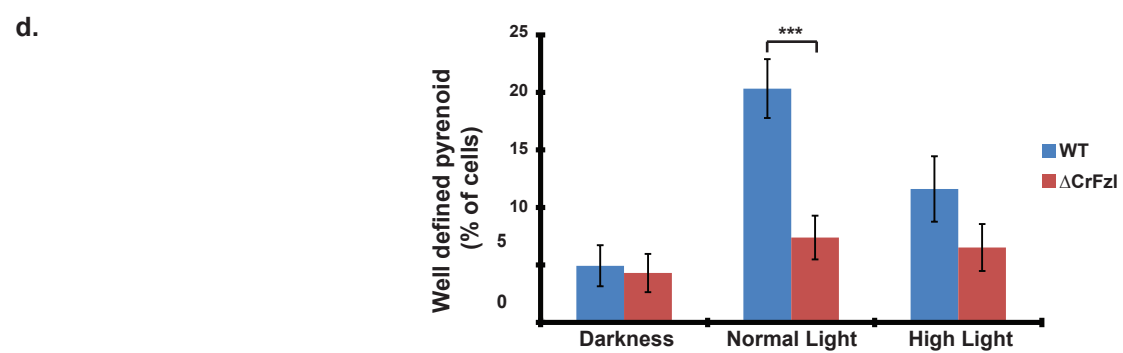

Supplement: S3 Fig — (a) Top panel. Fluorescence microscopy of wild-type and ΔCrFzl #1 chloroplasts after 6 hours of normal light (NL). Chlorophyll autofluorescence (Chl) is used to analyze chloroplast morphology. Scale bar: 2 μm. Bottom panel. Large fluorescence microscopy field of wild-type and ΔCrFzl #1 chloroplasts after 6 hours of normal light (NL) or high light (HL). Chlorophyll autofluorescence (Chl) is used to analyze chloroplast morphology. Scale bar: 5 μm. (b) Top 6 panels. Fluorescence microscopy of ΔCrFzl #1 cells after 6 hours of light stress. Images were taken with wavelength/filter sets used for detection of DAPI, YFP, CFP and chlorophyll (Chl). Scale bar: 5 μm. Arrowheads indicate auto-fluorescent dots detected with the DAPI, YFP and CFP filter sets. Bottom 4 panels. Fluorescence microscopy of ΔCrFzl #1 cells after 6 hours of light stress. Images were taken with wavelength/filter sets used for detection of GFP and chlorophyll (Chl). Scale bar: 5 μm. Arrowheads indicate auto-fluorescent dots detected with the GFP filter set. (c) Electron micrographs of wild-type and ΔCrFzl #1 cells exposed to normal light (left panel) and high light (right panel). cp: chloroplast; p: pyrenoid; arrows/v: vesicles; asterisks: extra-pyrenoidal starch granules. Scale bar: 2 μm. (d) Percentage of cells displaying a normal pyrenoid structure in wild-type and ΔCrFzl #1 cells during darkness, normal and high light conditions. Error bars represent the s.e.m. from the number of analyzed cells in WT Darkness: 71; WT NL: 91; WT HL: 56; ΔCrFzl Darkness: 93; ΔCrFzl NL: 88; ΔCrFzl HL: 69. ** p < 0,05. *** p < 0,005 (Student test). (PDF) [file pgen.1008047.s003.pdf]

a.

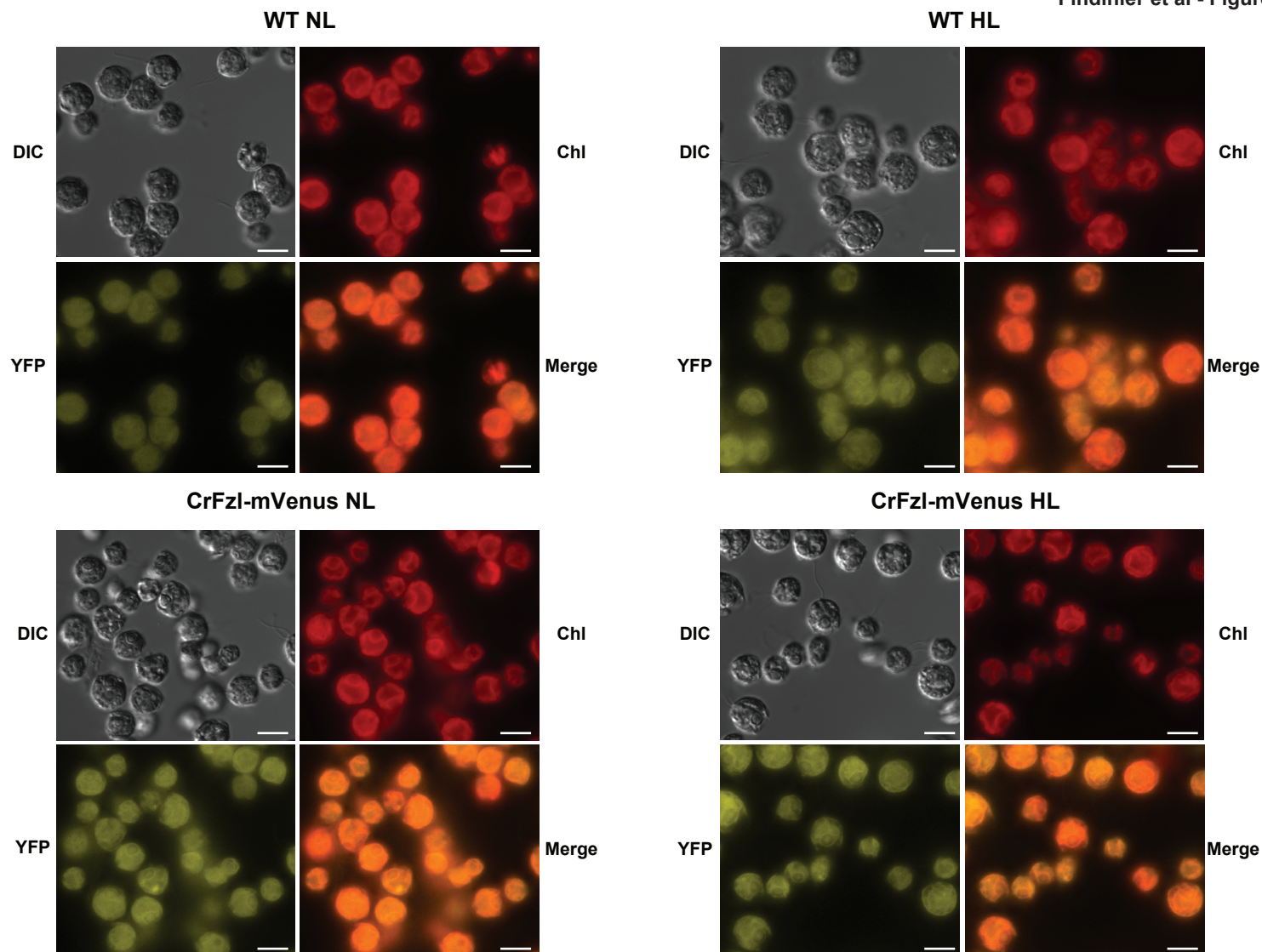

b.

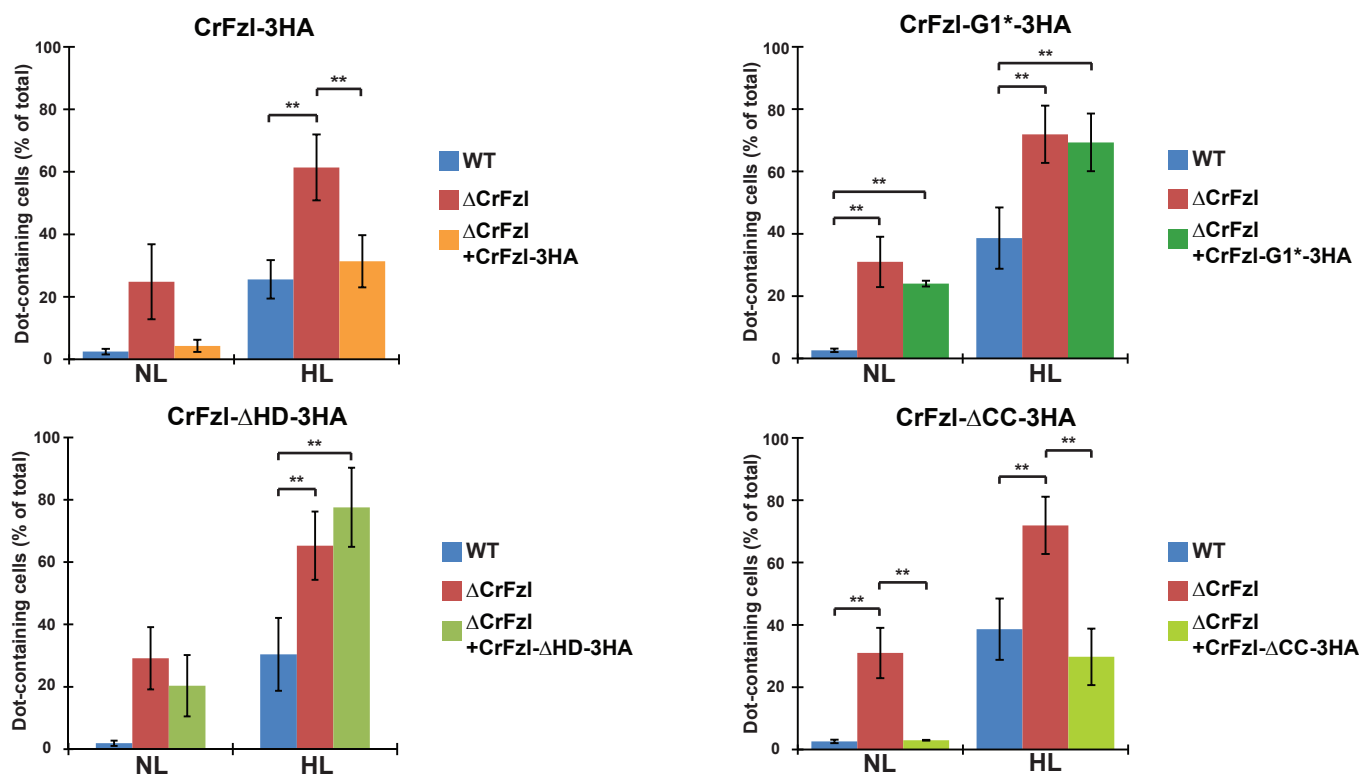

c.

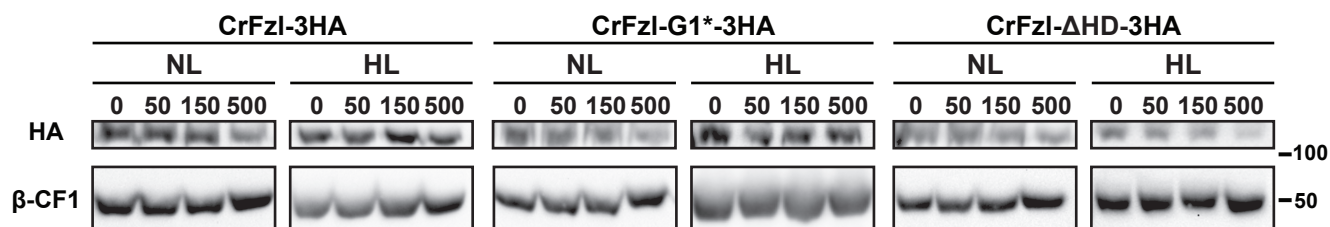

Supplement: S4 Fig — (a) Fluorescence microscopy of cells from the wild-type (top) and a ΔCrFzl strain expressing the CrFzl-mVenus construct (bottom) after growth in normal (NL, left) and high light (HL, right) conditions. Chlorophyll autofluorescence is used as a chloroplast marker. Scale bar: 5 μm. (b) Percentage of cells with fluorescent dots in normal (NL) and high light (HL) conditions in the wild-type (blue), the ΔCrFzl (red) and the ΔCrFzl strains expressing CrFzl-3HA (top left, orange), CrFzl-G1*-3HA (top right, dark green), CrFzl-ΔHD-3HA (bottom left, green) and CrFzl-ΔCC-3HA (bottom right, light green). Error bars represent the s.d. from three independent experiments. ** p < 0,05. *** p < 0,005 (Student test). (c) Western blot analysis of CrFzl-3HA, CrFzl-G1*-3HA and CrFzl-ΔHD-3HA in thylakoid fractions washed with 0, 50, 150 and 500 mM NaCl after 6 hours of normal (NL) or high light (HL) treatment. Loadings were adapted to reach similar amounts of β-CF1. Variations in CrFzl-HA in thylakoid washed with 50 and 150 mM NaCl are much less significant than at 500 mM and were then not shown in Fig 5f. (PDF) [file pgen.1008047.s004.pdf]

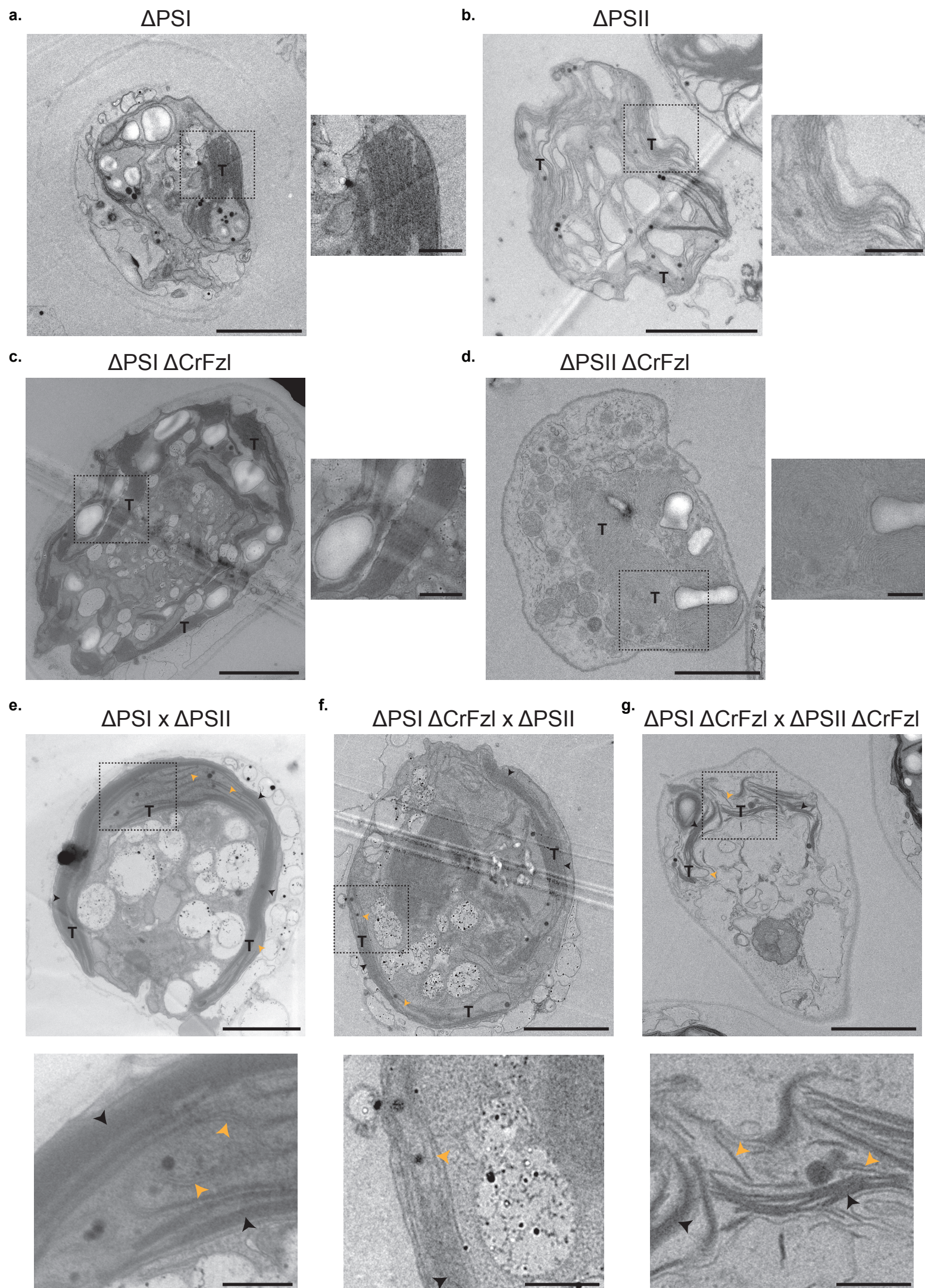

Supplement: S6 Fig — (a-d) Electron micrographs of ΔPSI (a), ΔPSII (b), ΔPSI ΔCrFzl (c) and ΔPSII ΔCrFzl (d) gametic cells. Thylakoids are annotated with a T and the dashed square delimits the enlarged area on the right panel. Scale bar, left panel: 2 μm; right panel: 500 nm. (e-g) Electron micrographs of zygotes from ΔPSI x ΔPSII (e), ΔPSI ΔCrFzl x ΔPSII (f), ΔPSI ΔCrFzl x ΔPSII ΔCrFzl (g) crosses. Thylakoids are annotated with a T and the dashed square delimits the enlarged area in the bottom panel. Black and orange arrowheads point to stacked and loose thylakoids, respectively. Scale bar, top panel: 2 μm; bottom panel: 500 nm. Two more cells of each kind of gamete and zygote are shown in Fig 7 and S7 Fig. (PDF) [file pgen.1008047.s006.pdf]

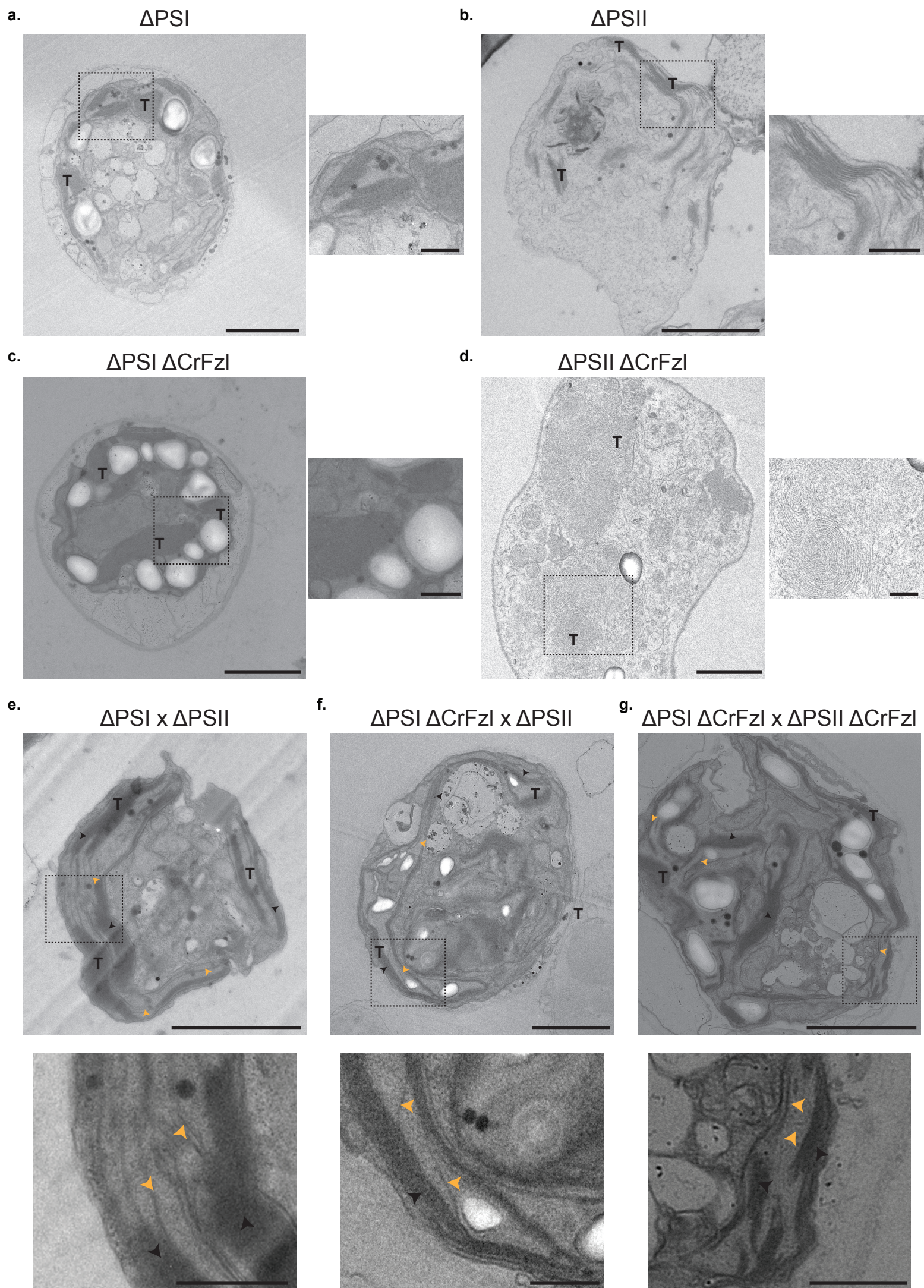

Supplement: S7 Fig — (a-d) Electron micrographs of ΔPSI (a), ΔPSII (b), ΔPSI ΔCrFzl (c) and ΔPSII ΔCrFzl (d) gametic cells. Thylakoids are annotated with a T and the dashed square delimits the enlarged area on the right panel. Scale bar, left panel: 2 μm; right panel: 500 nm. (e-g) Electron micrographs of zygotes from ΔPSI x ΔPSII (e), ΔPSI ΔCrFzl x ΔPSII (f), ΔPSI ΔCrFzl x ΔPSII ΔCrFzl (g) crosses. Thylakoids are annotated with a T and the dashed square delimits the enlarged area in the bottom panel. Black and orange arrowheads point to stacked and loose thylakoids, respectively. Scale bar, top panel: 2 μm; bottom panel: 500 nm. Two more cells of each kind of gamete and zygote are shown in Fig 7 and S6 Fig. (PDF) [file pgen.1008047.s007.pdf]
