# Supplementary material for: The dynamin-like protein Fzl promotes thylakoid fusion and resistance to light stress in Chlamydomonas reinhardtii
Source: PLoS Genet. 2019 Mar 15;15(3):e1008047. doi: 10.1371/journal.pgen.1008047 (PMC6436760; doi:10.1371/journal.pgen.1008047)
Supplement: S5 Fig — Two additional crosses (mating 2 and 3) are represented. (PDF) [file pgen.1008047.s005.pdf]

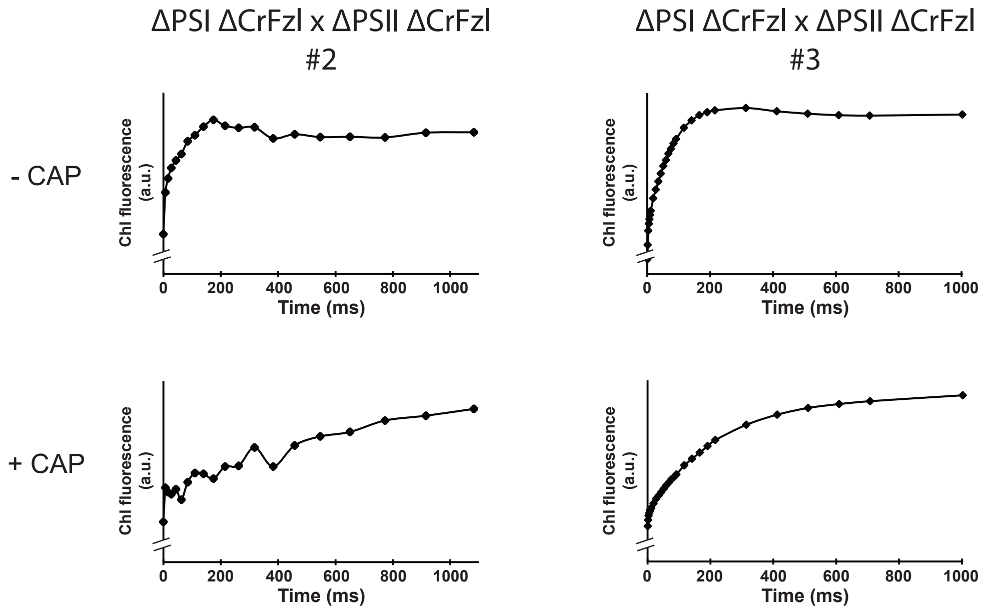
ΔPSI ΔCrFzl x ΔPSII ΔCrFzl
#2
ΔPSI ΔCrFzl x ΔPSII ΔCrFzl
#3
- CAP
+ CAP
Chl fluorescence
(a.u.)
Time (ms)
0
200
400
600
800
1000
