# Supplementary material for: The dynamin-like protein Fzl promotes thylakoid fusion and resistance to light stress in Chlamydomonas reinhardtii
Source: PLoS Genet. 2019 Mar 15;15(3):e1008047. doi: 10.1371/journal.pgen.1008047 (PMC6436760; doi:10.1371/journal.pgen.1008047)
Supplement: S1 Table — The total number of gametes (ΔPSI; ΔPSII; ΔPSI ΔCrfzl; ΔPSII ΔCrfzl) and zygotes (ΔPSI X ΔPSII; ΔPSI ΔCrfzl X ΔPSII; ΔPSI ΔCrfzl X ΔPSII ΔCrfzl) analyzed by electron microscopy is indicated. All gametes with the PSI mutation (ΔPSI and ΔPSI Δfzl) showed highly stacked thylakoids whereas those with the PSII mutation (ΔPSII and ΔPSII Δfzl) had less stacked thylakoids. In addition to being less stacked, the thylakoids from PSII Δfzl cells were systematically curved and often organized in fingerprint-like structures. All zygotes expressing Fzl (ΔPSI X ΔPSII and ΔPSI Δfzl X ΔPSII) had highly and less stacked thylakoids in continuity and organized as a proper network. In contrast, zygotes that do not express Fzl (ΔPSI Δfzl X ΔPSII Δfzl) had disorganized thylakoid networks with highly and less stacked thylakoids there were not in continuity. (PDF) [file pgen.1008047.s008.pdf]

	ΔPSI	ΔPSII	ΔPSI ΔCrfzl	ΔPSII ΔCrfzl	ΔPSI X ΔPSII	ΔPSI ΔCrfzl X ΔPSII	ΔPSI ΔCrfzl X ΔPSII ΔCrfzl
Total	**14**	**6**	**34**	**12**	**23**	**4**	**10**
Highly stacked thylakoids only	**14**	0	**34**	0	0	0	0
Less stacked thylakoids only	0	**6**	0	**12**	0	0	0
Curved thylakoids	0	0	0	**12**	0	0	0
Fingerprints thylakoids	0	0	0	**9**	0	0	0
Highly + Less stacked with continuity – well organized	0	0	0	0	**23**	**4**	0
Highly + Less stacked without continuity - Disorganized	0	0	0	0	0	0	**10**

Supplemental Table 1: Cells and associated phenotypes analyzed in the thylakoid fusion mating assay.

The total number of gametes (ΔPSI; ΔPSII; ΔPSI ΔCrfzl; ΔPSII ΔCrfzl) and zygotes (ΔPSI X ΔPSII; ΔPSI ΔCrfzl X ΔPSII; ΔPSI ΔCrfzl X ΔPSII ΔCrfzl) analyzed by electron microscopy is indicated. All gametes with the PSI mutation (ΔPSI and ΔPSI Δfzl) showed highly stacked thylakoids whereas those with the PSII mutation (ΔPSII and ΔPSII Δfzl) had less stacked thylakoids. In addition to being less stacked, the thylakoids from PSII Δfzl cells were systematically curved and often organized in fingerprint-like structures. All zygotes expressing Fzl (ΔPSI X ΔPSII and ΔPSI Δfzl X ΔPSII) had highly and less stacked thylakoids in continuity and organized as a proper network. In contrast, zygotes that do not express Fzl (ΔPSI Δfzl X ΔPSII Δfzl) had disorganized thylakoid networks with highly and less stacked thylakoids there were not in continuity
