# Supplementary material for: The dynamin-like protein Fzl promotes thylakoid fusion and resistance to light stress in Chlamydomonas reinhardtii
Source: PLoS Genet. 2019 Mar 15;15(3):e1008047. doi: 10.1371/journal.pgen.1008047 (PMC6436760; doi:10.1371/journal.pgen.1008047)
Supplement: S2 Table — (PDF) [file pgen.1008047.s009.pdf]

Primer name	Primer sequence	Usage
**CrFZL_ATG_For**	5'-CATATGATCGATAAGCTTGATATCGAATTCATGCAGCATTCAGCGAACGCAG-3'	Subcloning of CrFzl locus into pSL26 plasmid for HA-tagging
**CrFZL_ATG_Rev**	5'-AGGTTGCGGCTCAGTGCATGCGCCATGCCGGTCG-3'	
**CrFZL_STOP-HA_For**	5'-TACCAGTATCCATTGGGAAAGACGGGTATGCATTCC-3'	
**CrFZL_STOP_HA_Rev**	5'-AGTCGGGCACGTCGTAGGGGTATCGCGAGTACTCCAGGTTGGCGGT-3'	
**pLM_FZL_For**	5'-ACTGCTACTCACAACAAGCCCAGTTATGCAGCATTCAGCGAACGCAG-3'	Subcloning of CrFzl locus into pLM_005_CrVenus plasmid
**pLM_FZL_Rev**	5'-CGCCGGAGCCACCCAGATCTCCGTTGTACTCCAGGTTGGCGGTC-3'	
**HygFor**	5'-GACTGGAAAGCGGGCAGTGA-3'	Amplification of Hygromycin resistance cassette
**HygRev**	5'-AGACGGGCAGGTGTGTGCTC-3'	
**Hyg_500_Rev**	5'-GGAAGCGGACCGAGGACTTC-3'	PCR screening of CRISPR/Cas9 generated clones.
**Pre-G1_For**	5'-GCTCACGGACCAGGGTAAGAGCATACCGGTGCTGGCGAAGGAG-3'	Mutagenesis of CrFzl GTPase domain with the subsitution K446M
**Pre-G1_Rev**	5'-ATGACGGAGCTCATGCCGCTGTTGAACTCGC-3'	
**Post-G1_For**	5'-TCAACAGCGGCATGAGCTCCGTCATCAACGC-3'	
**Post-G1_Rev**	5'-CATGGAGACCGGAGTGCTTAAATCACCGGTGTGAAGTGCGACTTGGGG-3'	
**Pre-TM_For**	5'-ATAGGCGGGCGTGCGCAATGGCCAACGCGTTTGTTTGCGTTTG-3'	Removal of CrFzl predicted hydrophobic domains
**Pre-TM_Rev**	5'-CCTTGAGATCGGCGCGCCGCTGCTGCAGCTCGGTGTC-3'	
**Post-TM_For**	5'-TGGACACCGAGCTGCAGCAGCGGCGCGCCGATCTCAAG-3'	
**Post-TM_Rev**	5'-GCACCAATCATGTCAAGCCTCAGCACGCGTATTTAAATTACGTACCTGCAGGTCGCG-3'	
**CC_Rev**	5'-AGTCGGGCACGTCGTAGGGGTATCGCGAGTAGTCCGACTTGCGCGC-3'	Removal of CrFzl predicted Coiled coil domain

Supplemental Table 2: Primers used in this study
