# Supplementary material for: The dynamin-like protein Fzl promotes thylakoid fusion and resistance to light stress in Chlamydomonas reinhardtii
Source: PLoS Genet. 2019 Mar 15;15(3):e1008047. doi: 10.1371/journal.pgen.1008047 (PMC6436760; doi:10.1371/journal.pgen.1008047)
Supplement: S3 Table — (PDF) [file pgen.1008047.s010.pdf]

**CC-4533 (WT)**	***cw15 mt-***	**[27]**
**CrFzl-HA #1**	*cw15 CrFzl-3HA::aphVIII mt-*	This study
**CrFzl-HA #2**	*cw15 CrFzl-3HA::aphVIII mt-*	This study
**cw15 CrFzl-3HA (subfractionation)**	*cw15 CrFzl-3HA::aphVIII mt-*	This study
**Free mVenus**	*cw15 mVenus::aphVIII mt-*	This study
**CrFzl-mVenus**	*cw15 CrFzl-mVenus::aphVIII mt-*	This study
**cw15.J3 (WT)**	*cw15 mt-*	[56]
**ΔCrFzl CLiP (LMJ.RY0402.175738)**	*cw15 CrFzl::aphVIII mt-*	[27]
**ΔCrFzl #1**	*cw15 CrFzl::aph7’’ mt-*	This study
**ΔCrFzl #2**	*cw15 CrFzl::aph7’’ mt-*	This study
**ΔCrFzl #3**	*cw15 CrFzl::aph7’’ mt-*	This study
**ΔCrFzl #1 + CrFzl-mVenus**	*cw15 CrFzl::aph7’’ CrFzl-mVenus::aphVIII mt-*	This study
**ΔCrFzl #1 + CrFzl-3HA**	*cw15 CrFzl::aph7’’ CrFzl-3HA::aphVIII mt-*	This study
**ΔCrFzl #1 + CrFzl-G1* -3HA**	*cw15 CrFzl::aph7’’ CrFzl-G1* -3HA::aphVIII mt-*	This study
**ΔCrFzl #1 + CrFzl-ΔHD -3HA**	*cw15 CrFzl::aph7’’ CrFzl-ΔHD -3HA::aphVIII mt-*	This study
**ΔCrFzl #1 + CrFzl-ΔCC-3HA**	*cw15 CrFzl::aph7’’ CrFzl-ΔCC-3HA::aphVIII mt-*	This study
**F15 (ΔPSI)**	*nit1 nit2 tab1 mt+*	[38]
**Fud34**	*nit1 nit2 psbC mt+*	[40]
**Fud34.10- (ΔPSII)**	*psbC mt-*	This study
**ΔPSI ΔCrFzl**	*tab1 CrFzl::aph7’’ mt+*	This study
**ΔPSII ΔCrFzl**	*psbC CrFzl::aph7’’ mt-*	This study

Supplemental Table 3: Strains used in this study
